# Dynamic Mechanisms of Neocortical Focal Seizure Onset

**DOI:** 10.1371/journal.pcbi.1003787

**Published:** 2014-08-14

**Authors:** Yujiang Wang, Marc Goodfellow, Peter Neal Taylor, Gerold Baier

**Affiliations:** 1 Manchester Interdisciplinary Biocentre, The University of Manchester, Manchester, United Kingdom; 2 School of Computing Science, Newcastle University, Newcastle upon Tyne, United Kingdom; 3 College of Engineering, Mathematics and Physical Sciences, University of Exeter, Exeter, United Kingdom; 4 Cell and Developmental Biology, University College London, London, United Kingdom; University of Pittsburgh, United States of America

## Abstract

Recent experimental and clinical studies have provided diverse insight into the mechanisms of human focal seizure initiation and propagation. Often these findings exist at different scales of observation, and are not reconciled into a common understanding. Here we develop a new, multiscale mathematical model of cortical electric activity with realistic mesoscopic connectivity. Relating the model dynamics to experimental and clinical findings leads us to propose three classes of dynamical mechanisms for the onset of focal seizures in a unified framework. These three classes are: (i) globally induced focal seizures; (ii) globally supported focal seizures; (iii) locally induced focal seizures. Using model simulations we illustrate these onset mechanisms and show how the three classes can be distinguished. Specifically, we find that although all focal seizures typically appear to arise from localised tissue, the mechanisms of onset could be due to either localised processes or processes on a larger spatial scale. We conclude that although focal seizures might have different patient-specific aetiologies and electrographic signatures, our model suggests that dynamically they can still be classified in a clinically useful way. Additionally, this novel classification according to the dynamical mechanisms is able to resolve some of the previously conflicting experimental and clinical findings.

## Author Summary

According to the WHO fact sheet, epilepsy is a neurological disorder affecting about 50 million people worldwide. Even today 30% of epilepsy patients do not respond well to drug therapies. Neocortical focal epilepsy is a particular type of epilepsy in which drug treatments fail and surgical success rate is low. Hence, research is essential to improve the treatment of this type of epilepsy. Recent advances in brain recording methods have led to new observations regarding the nature of neocortical focal epilepsy. However, some of the observations appear to be contradictory. Here, we develop a computational modelling framework that can explain the different observations as different aspects of possible mechanisms that can all lead to seizure onset. Specifically, we classify three main conditions under which focal seizure onset can happen. This classification is clinically important, as our model predicts different treatment strategies for each class. We conclude that focal seizures are diverse, not only in their electrographic appearance and aetiology, but also in their onset mechanism. Combined multiscale recordings as well as stimulation studies are required to elucidate the onset mechanism in each patient. Our work provides the first classification of possible onset mechanism.

## Introduction

Neocortical focal seizures are episodes of pathological brain activity that appear to originate from spatially localised regions of the neocortex. The classical understanding of such seizures is that localised pathological tissue generates epileptic discharges (epileptogenic zone [1]), which subsequently recruit connected tissue, resulting in an epileptic seizure. Hence, the removal of the epileptogenic zone would result in seizure freedom [1]. Such a view is particularly applicable to focal epilepsy patients with e.g. cortical dysplasia, where a clearly localised anatomical abnormality of the cortex is present.

However, the classical understanding of neocortical focal seizures has not remained unchallenged, especially when treating patients without any clearly localised anatomical abnormalities. For instance, it is proposed that instead of a localised region of pathological tissue, an epileptogenic network [2]–[4] could underlie the generation of focal seizures. The spatial extent, and the participating regions of such a network are not yet clearly identified. Some indication is provided by the work of Stead *et al*. (2010) and Schevon *et al*. (2008), who report the recording of highly spatially localised epileptiform activity on the scale of cortical columns [4], [5]. Such electrographic activities, termed “microseizures” [4], [5], were recorded more frequently and for longer durations near the seizure onset zone [4]. Interestingly microseizures were also observed in non-epileptic control subjects, albeit in fewer locations and occurring less frequently than in epilepsy patients [4]. The authors hence proposed the hypothesis that “pathological microdomains” (i.e. microdomains that are able to generate and sustain isolated epileptiform hyperactivity states) might be found in healthy brains without leading to seizure onset. However, when occurring with sufficient density in one spatial area, they can form an epileptogenic network causing focal seizure onset from that area.

An alternative mechanism underlying (focal) seizure onset is proposed on the macroscopic scale. Badawy *et al*. (2009) demonstrated that the motor threshold of drug naive focal epilepsy patients decreased up to 24 h before a seizure on the ipsilateral side to the seizure focus [6]. A similar study using patients with mesial temporal lobe epilepsy also hints at an elevated motor cortex excitability preceding the seizure onset [7]. Hence, a change in overall cortical excitability has been suggested as a mechanism for focal seizure onset [8], [9]. This hypothesis is in line with the long-standing concept that seizures are a consequence of changing excitability of the brain [10]. However, the mechanism by which this general increased excitability over large parts of the cortex leads to focal onset dynamics is not specified.

An essential point of recent debate that is not explicit in either of the above suggestions of focal seizure onset mechanisms concerns the mechanisms of seizure recruitment and propagation. Based on the observation of single unit activity in human focal onset seizures, Truccolo *et al*. (2011) proposed that the recruitment process is “highly heterogeneous, not hypersynchronous, suggesting complex interactions among different neuronal groups even at the spatial scale of small cortical patches” [11]. In contrast, Schevon *et al*. (2012) suggests that seizure propagation is a well-structured process, where the recruitment progresses as a smooth wavefront. Recruited tissues show a synchronous firing activity that is phase locked to the local field potential.

It becomes clear that focal seizure onset and recruitment is still far from understood, and that prevailing hypotheses and observations lack a unifying framework in which they can be tested and analysed. In order to achieve this, we turn to mathematical models of cortical spatio-temporal dynamics. Traditionally, two types of models have been used: (i) Continuum models (e.g. [12], [13]) or neural field models (see [14], [15] for reviews) treat cortical tissue as a homogeneous continuous medium. The spatial extent often ranges from a few millimetres to a few centimetres [16], [17]. Pattern formation and travelling waves of activity have been studied extensively in these systems (see [18], [19] for reviews). Such spatio-temporal patterns have been related to epileptic activity. For example [17], [20] model the recruitment and propagation of a focal onset seizure as a propagating wave over a continuous medium. (ii) Network models treat the cortex as a connected network of cortical units (nodes), where often nearest neighbour, random, small-world or hierarchical connectivities are used. Depending on the definition of the network nodes, these models are used across all scales from local populations of neurons [21] to the whole brain level [22], [23]. Network based models have investigated how network structures impact seizure synchronisation dynamics [23]–[25], seizure frequency [26], or the spread of seizure activity from an epileptic focus [27],[28]. To specifically model the mesoscopic epileptic dynamics of extended cortical tissue, [29] suggests arranging coupled units of neural mass models (see [30] for review) as a sheet. Similarly, [31], [32] arrange neural mass models according to the tessellated surface of the brain and coupled neighbours to simulate scalp and intracerebral dynamics of focal seizures. Such an approach, although technically a network approach, can approximate the behaviour of continuum models (compare [29] and [20]). Recently, [33] also relates a network of mass models to an equivalent field model directly.

However, the connectivity in realistic cortical tissue appears to require a combination of both continuum and network approaches. Connections to nearby neighbours are very dense [34], such that it approaches the continuum case. Nevertheless structured long-range connections can form a complex network that is best described by a networks approach [35]. Hence, to describe the mesoscopic scale of the cortex, combinations of both network and continuum approaches have also been suggested, e.g. including heterogeneous long-range connections in neural field models (see for example [36]–[39]). Starting from a network perspective, Voges *et al*. (2010) propose to use a network model that includes dense local connections, approximating the continuum case, and incorporate remote excitatory connections that bridge distances of several millimetres [35]. The remote connections are furthermore structured and tend to target remote clusters or patches.

In this work, we advance upon previous spatio-temporal network models of cortical tissue on the mesoscopic scale and use a dense array of cortical units (cortical columns) that reflect the activity of local neuronal populations. For connectivity between the units we use the suggestion in [35] and incorporate dense local connections as well as patchy remote connections. This model has the advantage of combining both types of modelling approaches and thereby we create a spatially hierarchical model to study multi-scale dynamics.

Using this model, we investigate the dynamical mechanisms leading to the observation of focal onset seizure activity. We find that three different classes of dynamical mechanisms are compatible with a focal onset of an abnormal rhythm. Each of these classes show particular distinguishing features in terms of their dynamics and response to stimulation. Furthermore, they suggest alternative treatment strategies that could provide the basis to improve treatment options for patients in the future.

## Model

### Model of a cortical mini-column

We commence by defining the smallest unit in our model: the cortical minicolumn. This choice is based on the highest spatial resolution of the clinical observations with which we compare the model output. To reflect the electric activity of a minicolumn, we use an established model of excitatory and inhibitory neural population activity: the Wilson-Cowan model [40].

**Figure 1:**
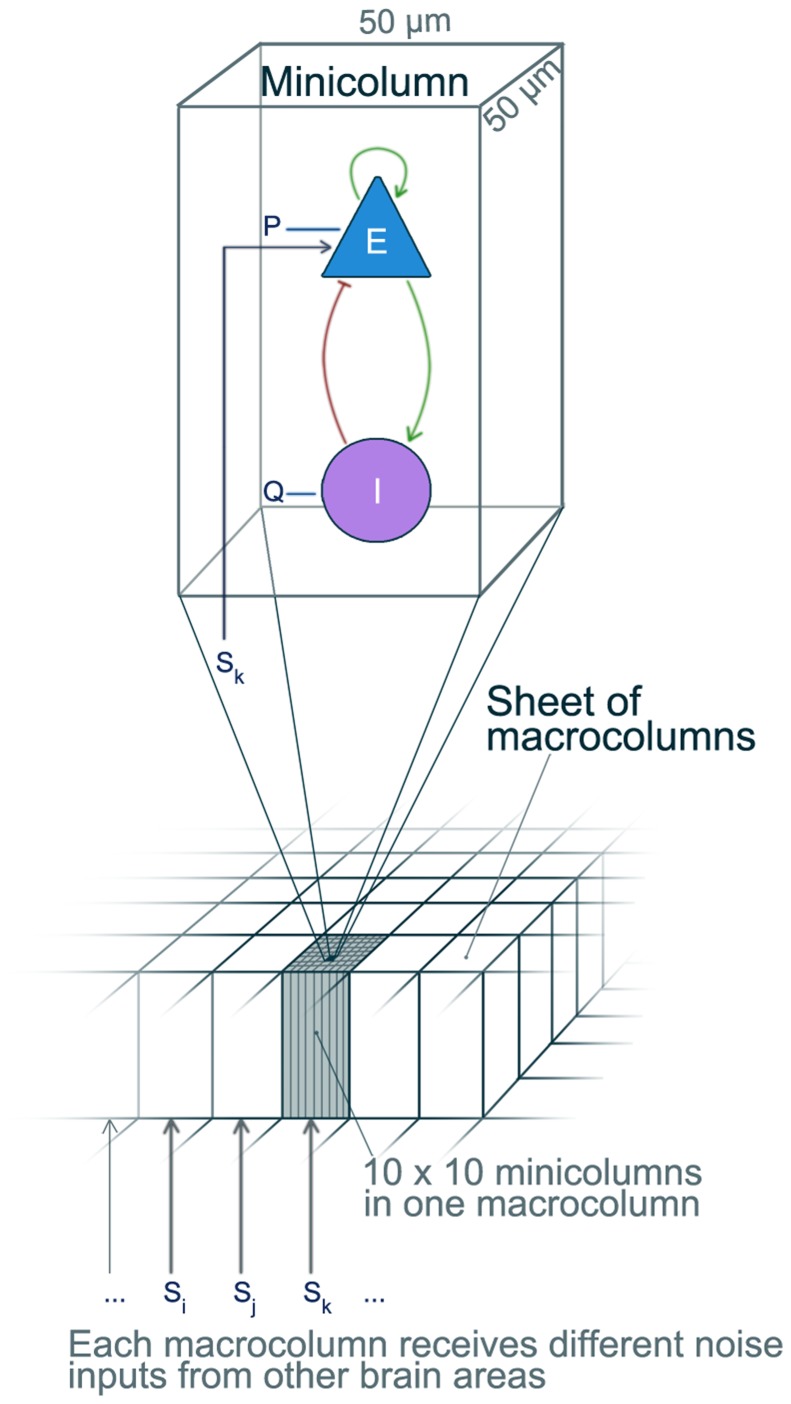
Schematic illustration of the structure of the model of a cortical sheet. The cortical sheet consists of an array of macrocolumns, each containing 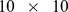 minicolumns. Each minicolumn within a macrocolumn is modelled by a Wilson-Cowan 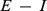 unit (Eqn. 1) and receives the same noise input representing input from other brain areas.

This model expresses the percentage firing activity in an excitatory (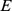) and an inhibitory (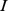) neural population over time. It assumes that the 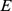 and 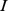 populations are coupled to each other and that the inputs to a target population sum together and influence the firing activity of this population. We use such a coupled 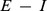 unit to represent a single cortical minicolumn (see Fig. 1).

The equations for our 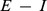 model are: 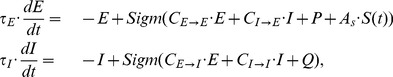
(1)where 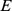 is the fractional firing activity in the excitatory population; 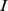 is the fractional firing activity in the inhibitory population; 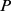 and 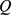 denote the basal activity level of the excitatory and inhibitory populations, respectively; 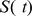 is the noise input to the excitatory population (e.g. subcortical input) with 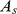 as the coupling strength of the noise input; and the connectivity constants 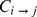 (with 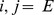 or 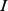) regulate the coupling strength between the populations.


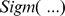
 is a sigmoid function, which derives from a distribution of firing thresholds in the underlying neural population [40]. It is defined as 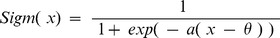, where 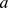 is the steepness of the sigmoid and 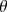 is the offset (in 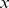) of the sigmoid. We fix the sigmoid parameters (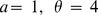) following previous work [41], as variations in the other parameters 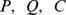 effectively result in a change of the sigmoid shape.

The Wilson-Cowan model has been subject to extensive studies in the last decades [42]–[44]. The slightly simplified version in Eqn. 1 (see also [45]) was shown to maintain the same bifurcation structures as the original model [41]. The simplification removed the bracket of 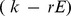 (or 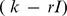) that the sigmoid was multiplied by in the original equations. Mathematically, the term has little impact on the dynamics. It is essentially rescaling the phase space and parameter space.

### Model of a cortical sheet

In order to model cortical tissue, we connect an array of minicolumn units to form a cortical sheet (also referred to simply as a sheet). We also refer to each of these minicolumn units simply as units. This formulation assumes an effectively two dimensional structure for the cortex. In reality, there is interplay between the three dimensional cortex, subcortical structures and other brain regions. However by making the simplifying assumption above, these influences in brain dynamics are absorbed into the intrinsic parameters of a minicolumn. Similar approaches of modelling the cortex as a 2D sheet can be seen in [12], [15], [29], [46], [47].

As an approximation we assume all minicolumns to be 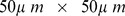 in size [48]. A macrocolumn is then formed by 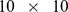 minicolumns, which agrees with the size suggested in [49]. Furthermore we investigate cortical sheets with 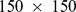 minicolumn units (i.e. 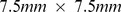, or 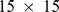 macrocolumns). Thus 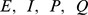 and 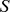 in Eqn. 1 become vectors of the length 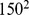; and the connectivities 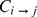 become matrices of the dimension 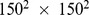. We limited the size of the sheet to 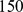 minicolumns in length, as we assume the mean activity of such a sheet reflects the signal recorded on a single ECoG electrode.

Each excitatory population is additionally driven by noise (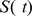) representing input from other unmodelled regions, e.g. subcortical input. The noise is the same within each macrocolumn in agreement with experimental findings and the definition of macrocolumns [48], [50]. We used noise values drawn from a standard normal distribution as input. The effective noise coupling strength is set to 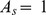. In this setting the system is not entirely dominated by the noise input but the noise influences the deterministic dynamics. Simulations of the system used a fixed step solver, with a stepsize of 2 ms. Qualitatively equivalent results are found for smaller stepsizes. Fig. 1 schematically summarises the model.

### Connectivity

**Figure 2:**
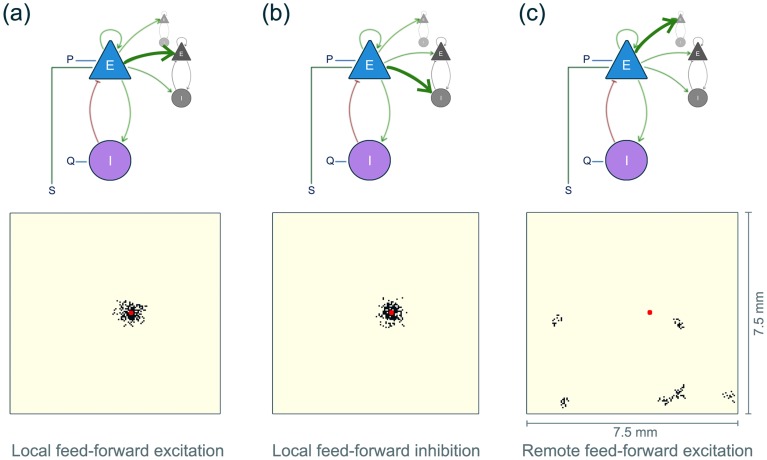
The connectivity in our model of a cortical sheet. Top row: schematic illustration of the three connection types (strong green arrow). The coupled 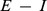 unit represents one cortical minicolumn. The dark grey unit represents a local neighbour and the light grey unit represents a remote unit. Bottom row: For the same exemplar minicolumn unit (indicated as a red dot) we show the units it projects to (black dots) under the indicated connectivity.

In the model we use three types of connections between minicolumns, based on the cortical connectivity suggested by Voges *et al*. (2010) [35]. All choices for parameters of the connectivity are also based on [35], where they are derived from tract tracing experiments in human cortical tissue.

(I) The first type consists of local excitatory connections, where each excitatory population of a minicolumn unit connects to the excitatory populations of neighbouring units in its immediate proximity (Fig. 2 (a), top). Here, each unit has a probability to connect to its neighbours that follows a Gaussian fall-off with distance. The standard deviation 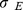 of the Gaussian is set to 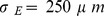, as within 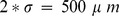 radius most local connections are found [35]. We furthermore do not allow for local excitatory connections beyond a radius 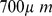 as these are incorporated into a specific longer range connectivity scheme, as described below. Fig. 2 (a, bottom) shows an example of one unit (red) and the neighbouring units (black) is sends local excitatory connections to. The connectivity matrix for the local excitatory connections is denoted 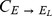, where each connection has the weight 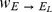 (subscript 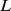 denoting local connections).

(II) The second type of connections is from the excitatory population of each unit to the inhibitory populations of close neighbours (Fig. 2 (b), top). We use the same algorithm and parameters as in (I) to generate these connections. Fig. 2 (b, bottom) shows an exemplary realisation of local inhibitory connections from one unit. We refer to the connectivity matrix for the local inhibitory connections as 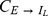, where each connection has the weight 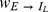.

(III) The third type are remote patchy overlapping connections from each excitatory population to excitatory populations at some distance (Fig. 2 (c)) [35]. All the parameters are following the suggestions in [35]. We generate 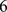 random patches for each macrocolumn and all minicolumns within the macrocolumn can connect to these patches with 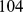 outgoing connections. This fulfils the suggested average ratio of 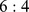 of local connections to remote connections [35]. Each patch consists of 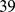 minicolumns (the patch radius is 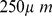, i.e. 5 minicolumns radius, i.e. 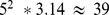 minicolumns) and is located within 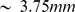 distance. Macrocolumns share 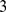 patches with one direct neighbouring macrocolumn, which can increase the distance between macrocolumn and target patch to more than 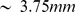. These parameters are in line with the suggested and experimental values listed in [35]. The algorithm that generates the remote connectivity matrix is described in Text S1. We call the connectivity matrix for the remote excitatory connections 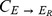, where each connection has the weight 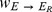, (subscript 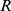 denoting remote connections).

The connectivity matrix 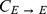 therefore consists of 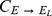, 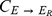 and the self-excitation value of each excitatory population on the diagonals (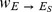, subscript 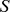 denoting self connection). Similarly 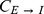 consists of 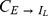 and the connection value of the 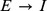 connection within the minicolumn unit on the diagonals (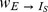). The other 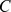 matrices are diagonal matrices only, as they are exclusively connections within a minicolumn. Long-range inhibition is not included, following [35].

In order to aid the understanding of the resulting connectivity being created by the aforementioned rules, Fig. S1 additionally show the in/out degree and the distance distributions of the local, as well as remote connections. Text S1 further explains the details of the connectivity.

A cortical sheet with toroidal boundaries was used in the construction of the connectivity matrices, following [35], to avoid boundary cut-off effects caused by lack of basal input due to lack of neighbours. Text S2 discusses in detail how different boundary conditions affect the model dynamics and we will show that all our presented results are not affected qualitatively by the choice of boundary conditions.

### Model parameters

The choice of model parameters for the isolated Wilson-Cowan unit was based on dynamical reasoning. The dynamics of a single minicolumn unit (in the following referred to as 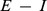 unit) has been subject to extensive studies. The invariant dynamic behaviour in an 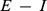 unit is limited to either a stable fixed point (node or focus) or a stable limit cycle, and two stable fixed points (see [41], [42] for details). As we are interested in the transition between fixed point and limit cycle we select model parameters in the vicinity of the transition to oscillations. Depending on the combined parameter variation, either a homoclinic or a Hopf bifurcation occurs. However the single 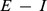 unit in model is incapable of oscillations, even with increased constant input 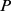. Text S3 shows details of the current parameter setting for a single 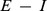 node.

Based on the dynamics of a single 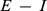 unit, the dynamics of the fully coupled sheet is classified as: (i) fixed point (corresponding to the lower fixed point in the 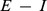 unit; the spatial average of the whole system does not show prominent regular oscillations over time); (ii) oscillation (corresponding to the limit cycle in the Wilson-Cowan unit; the spatial average of the whole system shows high amplitude oscillations over time); or (iii) bistability between fixed point and oscillatory state. Although the coupled Wilson-Cowan systems are known to show a complicated repertoire of oscillatory states (in term of regularity and phase relationships [43]), we do not sub-classify the oscillatory states further. The epileptic EEG or ECoG has a considerable noise component and is non-stationary such that a reliable classification from clinical data is challenging. Also, a theory of spatio-temporal patterns in large heterogenous networks of nonlinearly coupled nonlinear oscillators is lacking. However, it was shown previously that a combination of mathematical understanding of a single network unit and computational studies of the network can lead to improved understanding of clinically relevant phenomena (e.g. the generation of oscillatory afterdischarges in epileptogenic cortical tissue [29].

### Seizures in the model

In the clinical setting diverse waveforms can be observed in electrographic recordings of neocortical focal seizures. However, we seek a simplification of this situation in our model, which captures some essence of abnormal dynamics during seizures. We therefore focus on the existence of high-amplitude oscillations in the model output as representative of seizure activity, in contrast to a low-firing state, which is representative of “background” or inter-ictal activity. This idea follows previous modelling studies (for example [51]–[53] and references therein). The approach is further supported by the suggested clinical definition [54] of a seizure state as oscillations in unit firing, which are phase locked to high amplitude local field potential oscillations. The background state is characterised by irregular firing patterns, which do not correlate with any oscillations in the local field potential.

In a single unit, we shall hence identify the background with the fixed point. As the Wilson Cowan oscillator only has one limit cycle representing synchronous rhythmic firing activity on the local population level, we shall identify this limit cycle with the local seizure state. Our model is additionally capable of a third state: the permanently firing state (referred to as “upper fixed point” in the Wilson-Cowan model). This state is not identified with any clinically observable state, and we hypothesise that the parameter settings required to reach this third state do not play a functional role during focal-seizure onset.

In the simulated coupled sheet, we understand high-amplitude synchronous (plus minus phase shift) oscillations in firing and LFP over several connected units as the seizure core [54]. Hence, full recruitment will be understood if the whole sheet is in such a state, where all units are in a synchronous oscillatory state. Text S5 describes how we detect these full recruitments or localised non-recruiting seizure cores for each figure. Other types of oscillations (e.g. non-synchronous low amplitude oscillations, which could represent non-pathological oscillations) on the full-sheet level were not specifically identified or analysed.

The matlab code for the model is published online (ModelDB Accession number: 155565).

## Results

In a first step we focus on the mean-field dynamics of the model and how they vary due to changes in parameters. In subsequent sections we use this insight to investigate the spatio-temporal mechanisms by which focal onset seizures can occur. For each mechanism we summarise how it can be distinguished from other mechanisms, how they relate to clinical and experimental observations, and which treatment strategies could be effective.

### Mean-field dynamics

**Figure 3:**
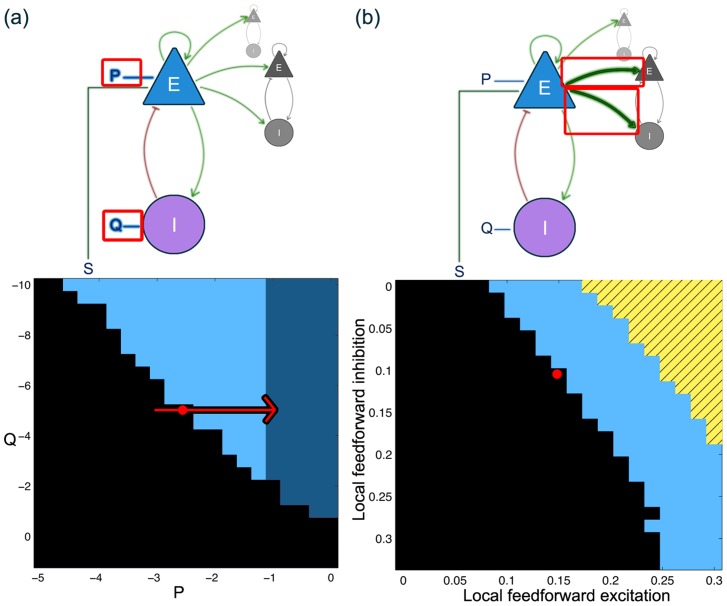
Mean-field behaviour of the whole system depending on P/Q (a) and feed forward excitation/inhibition 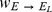/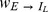 (b). Black indicates monostable background. Light blue indicates bistable oscillatory state and background state. Dark blue indicates monostable oscillatory state, and striped yellow indicates bistable background and upper fixed point. The red dot indicates the standard parameter setting used for the remaining manuscript, if not mentioned otherwise. Red arrow shows the parameter shift used for Fig. 4. Details of the parameters and the scan are described in Text S5.

To chart the dynamics of the model cortical sheet with respect to parameter changes, we focus initially on spatially homogeneous variations in the four parameters highlighted with red frames in the schematic in Fig. 3, i.e. 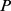, 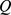, 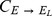 and 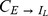. Fig. 3 demonstrates that there are large regions of parameters for which the system resides in the background state (black regions in Fig. 3), or oscillatory state (dark blue regions in Fig. 3). Additionally, in some parameter regions the oscillatory state can be found to be bistable to the background state (light blue regions in Fig. 3). A consequence of this is that a system in this parameter region can exhibit either background or oscillatory dynamics under the same parameter conditions. The transition from background to oscillatory activity is dependent upon all four scanned parameters. Pairwise scans of additional model parameters can be found in Text S4, which demonstrates that combinations of other parameters also give rise to background, oscillatory or bistable dynamics.

From the dynamical systems perspective it is often assumed that the epileptic brain resides in a parameter setting close to the onset of oscillations [51]. Hence, we selected one standard parameter set for our model in line with this idea, as indicated in Fig. 3 (a) and (b) by a red dot. This standard parameter set serves as our model interictal state, or monostable background state. Dynamically, the interictal state is a node and excitability can be detect for a range of stimuli in this state (data not shown). For an exemplary parameter change (red arrow in Fig. 3 (a)) we have also analysed the transitions in detail in a noise-free system. The monostable background state is the only stable fixed point in our system at 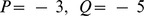. The onset of bistable high-amplitude oscillations occurs suddenly at 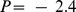. At the transition to monostable oscillations (at about 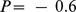), the background node ceases to exist and the oscillatory state becomes the only stable state. When changing 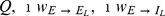 from the standard interictal parameter setting, similar transitions occur, only the background node remains stable and does not cease to exist.

Having demonstrated the effect of global parameter changes on the mean-field dynamics of the model, we proceed to examine the different ways in which transitions to seizure activity can occur spatio-temporally.

### Class I: Globally induced focal-onset rhythm

**Figure 4:**
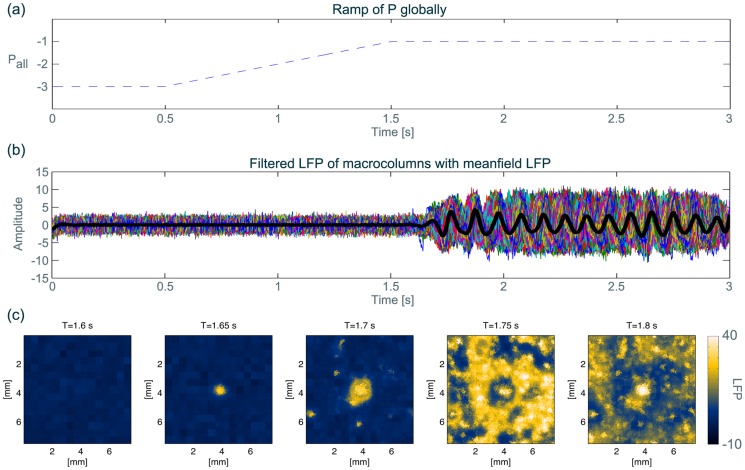
Input parameter 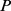 ramped homogeneously in all units, inducing focal onset of abnormal activity. (**a**) Ramp of 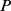 in all units over time. (**b**) Resulting dynamics of simulated LFP shown as the spatial average of macrocolumns. (**c**) Snapshots in time of the same time series in (b). In this example, a patch of tissue in the middle (see second snapshot) receives 1.5 times stronger feed forward excitation than the rest of the sheet. Video S1 shows the temporal evolution of another run of the same simulation conditions. Details of the parameter settings and changes are described in Text S5.

The parameter scans in the previous section imply that a slow parameter change that crosses from the background to the oscillatory region can induce a transition from background to seizure dynamics on the mean-field. An example of such a parameter ramp over time is indicated by the arrow in Fig. 3 (a) and in Fig. 4 (a). This suggestion follows a traditional modelling approach of seizures induced by *bifurcations* (see for example [55], [56], also c.f. [57]). In simulations of this scenario the onset of the abnormal rhythm is approximately simultaneous in all spatial locations, as the corresponding parameter 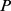 is modified simultaneously in all units across the sheet. In our case, the transition occurs at about 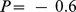 as a bifurcation from a node to an oscillatory state, where the onset of oscillation frequency and amplitude is sudden and discontinuous, and the node ceases to exist.

Using such a transition, we sought to establish whether the model can produce focal onset dynamics. Typically cortical tissue is not globally homogeneous. We therefore consider the impact of a locally altered region of model cortex, which is realised by assuming a local parameter heterogeneity in the model. We specify a patch in the middle of the model cortical sheet that receives increased feed-forward excitation. This heterogeneity is not visually detectable in the interictal state (see Fig. 4 (c), first panel). However, in a simulation with a parameter ramp as shown in Fig. 4 (a), the heterogeneous region displays an earlier response (Fig. 4 (c)). Dynamically, the earlier ignition of activity in the heterogeneity is due to the introduced difference in feed-forward excitation, which lowers the threshold beyond which oscillatory dynamics can ensue.

#### Dynamical onset mechanism

In this class of onset mechanism, the seizure onset is caused by a global parameter change in an extended piece of tissue. The global parameter change results in a bifurcation to global oscillatory dynamics in the mean-field. The focal onset is a consequence of an otherwise silent local tissue heterogeneity. The heterogeneity itself only reflects the global change first, but does not cause the transition. We refer to this mechanism as *globally induced focal onset seizures*.

#### Nature of the focus

The local tissue heterogeneity does not show abnormal background dynamics. Being closer to the oscillatory region in parameter space it could show enhanced response to stimulation compared to its surrounding in the interictal state. It could be detected by spatially resolved single pulse stimulation, for instance as showing larger amplitude responses or prolonged oscillatory transients, in agreement with [58].

#### Distinguishing features and propagation pattern

Following the focal onset the overall propagation pattern is not necessarily organised as a wavefront but can appear fairly heterogeneous depending on the sequence in which different parts of the tissue are ignited.

#### Clinical relevance

Our model predicts that in this class of onset mechanism, removal of the locally heterogeneous patch does not prevent the remaining model tissue from entering the seizure state. Resection of the apparent focus will only lead to an apparent focal onset from another region, e.g. a second region with increased susceptibility. A “secondary” seizure focus is indeed observed in some patients with focal seizures after surgery. The concept of secondary foci has been proposed by [1] in the context of coexisting seizure onset zones with different thresholds, although not explicitly conceptualised in the context of spatio-temporal dynamics. The global parameter shift required by this onset mechanism could potentially be observed via active stimulation. For instance Badawy *et. al* (2009) [6] show that the motor threshold decreases before seizure onset, indicating a change on the whole-brain level on a time scale of minutes to hours. Thus the risk of an imminent seizure could be assessed by monitoring response to active stimulation, e.g. the transcranial magnetic stimulation threshold [8], [9]. Our model also confirms that a global decrease in threshold can be detected by stimulation (Text S6, and Fig. S7).

#### Predicted responses to treatment

To causally prevent seizure onset the source of the global parameter shift needs to be identified and the course of the parameter shift altered. Global treatment methods which, increase the distance from the seizure threshold (such as anti-epileptic drugs) are expected to be successful in this class. A local treatment (e.g. tissue resection) only prevents seizure onset in the treated area but seizures are likely to continue, starting from a different part of the cortex. Local disruption of connectivity will not lead to seizure freedom.

### Class II: Globally supported focal-onset rhythm

Next we explore the mechanisms leading to focal onset rhythmic activity when the whole model cortical sheet is in the bistable background state. A bistable state has been proposed to underlie situations in which the transition to abnormal brain activity is not caused directly by global parameter changes (see e.g. [59]). It was postulated specifically as underlying the transition to epileptic seizures in the context of generalised [53], [60] and focal seizures [17], [28], [61].

In order to explore this scenario we prepare the cortical sheet in a global parameter setting of bistability. If the sheet is initiated in the background state, it will remain in the background state in the absence of strong perturbations. To initiate oscillatory activity, the background state can be disturbed in two possible ways: either by a short, temporary stimulus or by a persistent stimulus. We shall explore both perturbations in the following.

### Class IIa: Temporary stimulation trigger

We prepared the cortical sheet in the bistable background state by decreasing the feed-forward inhibition compared to the interictal parameter setting. Our choice to decrease feed-forward inhibition is inspired by the suggestion that a failure of inhibitory restraint [54] contributes to seizure onset. Equally, a change in 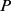, or other parameters could have been used.

**Figure 5:**
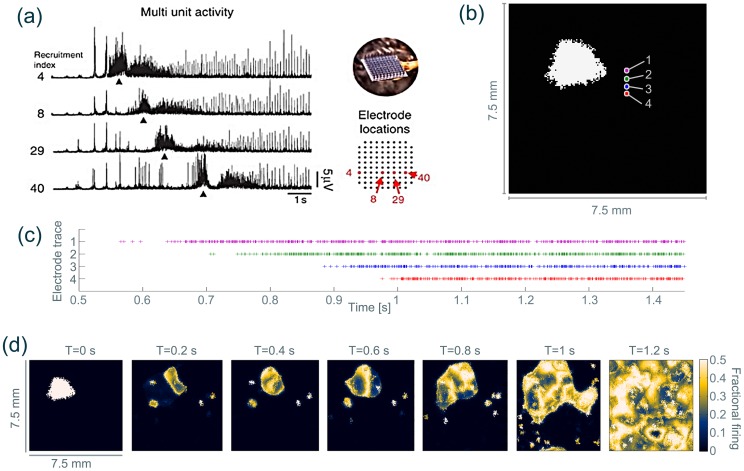
Propagating wave observed after a temporary local stimulus to a bistable sheet. (**a**) Multiunit firing as recorded in the human epileptic cortex in different microelectrodes. A propagating wavefront of increased multi-unit activity can be observed. (Reprinted by permission from Macmillan Publishers Ltd: Nat. Commun. [54], copyright 2012.) (**b**) Cortical sheet with units that receive a pulse stimulus marked in white. Marker numbers indicate the units used for the time series in (c). (**c**) Time series of firing activity of the 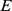 population for the four units marked with numbers in (b). (**d**) Snapshots of the firing activity of the 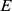 population of the sheet at different time points showing a propagating wavefront of recruitment into the oscillatory state. A video of the temporal evolution is shown in Video S2 (compare with Supplementary Video 5 in [54]). Parameter details are described in Text S5.

In our model, the bistable background state (dynamically also a node) does not show any obviously different dynamics compared to the monostable background state. However, when perturbed locally by a pulse-stimulus, the whole cortical sheet can transit to the co-existing oscillatory state. In Fig. 5 (c) and (d) we demonstrate how this transition unfolds in terms of spatial-temporal dynamics. After the stimulation, a subset of the stimulated units transits to the bistable oscillatory regime, which subsequently recruits neighbouring units into the oscillatory state. The recruitment in this connectivity parameter setting progresses as a wave, similar to the observation by Schevon *et al*. (2012) [54]. The comparison between clinical data and the simulation is invited in Fig. 5. In both cases the continuous progression of a wavefront of increased firing activity is observed.

In the current connectivity setting, heterogeneities in the propagation dynamics can also be observed. This is due to the heterogeneously created remote projections, which can support the activation of tissue at some distance from the primary recruitment site. A purely local connectivity creates an even propagation front (Text S7A, and Fig. S8 (a)), and a purely remote connectivity gives rise to stochastic patchy propagation (Text S7A, and Fig. S8 (b)). A mixture of propagation behaviours between these two extremes can be observed when a connectivity scheme that combines both features is used (as in Fig. 5 (d)).

To demonstrate that these findings are repeatable and reliable despite the noise input to the system, we scanned the recruitment speed after a (fixed) pulse stimulus for different values of 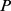 and 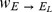 in and around the bistable parameter setting. Averaged over 5 trials using different noise input, little variation in recruitment speed due to noise was found for a fixed parameter set. However, recruitment speed did vary with the parameter settings (see Text S7B for details).

The stimulus size was also found to influence the recruitment, and a minimal stimulus size was found to exist depending on the parameter setting (Text S8A and Fig. S11). This means that a critical number of units have to be stimulated to induce the transition of the sheet to the seizure state, when it is bistable. This finding is potentially important for the clinical determination of the spatial extent of pathological stimuli in a patient-specific context.

#### Dynamical onset mechanism

In this section we have introduced a category where seizures are supported by a global bistability condition and initiated by a local temporary stimulus. We refer to this as *globally supported focal-onset seizures triggered by local pulse stimulation*. No parameter change is required to start the transition, but the temporal stimulus serves to switch from one stable attractor in state space to another.

#### Nature of the focus

The onset of the seizure rhythm is at the location of the trigger. In the clinical setting, a temporary trigger could be any (not necessarily pathological) localised input, which temporarily increases the activity of a patch of tissue (i.e. a “particular exogenous input” [6]). The focal-onset region could shift and change positions, e.g. when different external inputs cause the trigger to be in slightly different positions.

#### Distinguishing features and propagation pattern

In this category, the global bistability can be explicitly tested in experimental models, for instance, by local electrical stimulation. The seizure propagation dynamics in this category can be varied (ranging from an uniform wave front, to patchy activation).

#### Clinical relevance

The bistability of the surrounding can be reached for instance by a parameter change from the monostable to the bistable parameter region. During the parameter shift, the observable dynamics remains in the background. Such a global parameter change without noticeable behavioural or electrographic change is in agreement with the establishment of the peri-ictal state as suggested in [6], [8]. The peri-ictal state can be detected up to 24 h before focal onset seizures and is marked by increased cortical excitability following stimulation, but no seizure events [6]. In such a peri-ictal state, a trigger can elicit a full-recruitment seizure event. One candidate to be investigated for this kind of mechanism is reflex epilepsies which are characterised by precipitated seizures.

#### Predicted treatment

In this category, any measure to avoid the local trigger would serve to prevent seizure onset. As the input is only temporary and is not necessarily pathological (as in the case of reflex seizures), surgical removal of onset zone might not be indicated. Preventing the parameter changes leading to global bistability (e.g. using globally acting anti-epileptic drugs) would be expected to successfully prevent seizure onset. As shown in our parameter scans, many different combinations of parameter changes can lead out of the region of global bistability.

An alternative to drug therapy could be local counter stimulation in the case of a temporary trigger. If the trigger can be localised and detected early (in a closed-loop stimulation protocol), suppression of local seizure onset activity (e.g. by using high frequency stimulation [62]) can attenuate or prevent subsequent recruitment and spreading. We demonstrate in Text S9 that an appropriate counter stimulus delivered at the correct time after seizure onset can prevent the seizure from spreading. Finally, it should also be possible at any time during the seizure to give counter stimuli to abort the rhythm and recover a normal background dynamics if the bistability persists [61].

### Class IIb: Local persistent activity trigger

**Figure 6:**
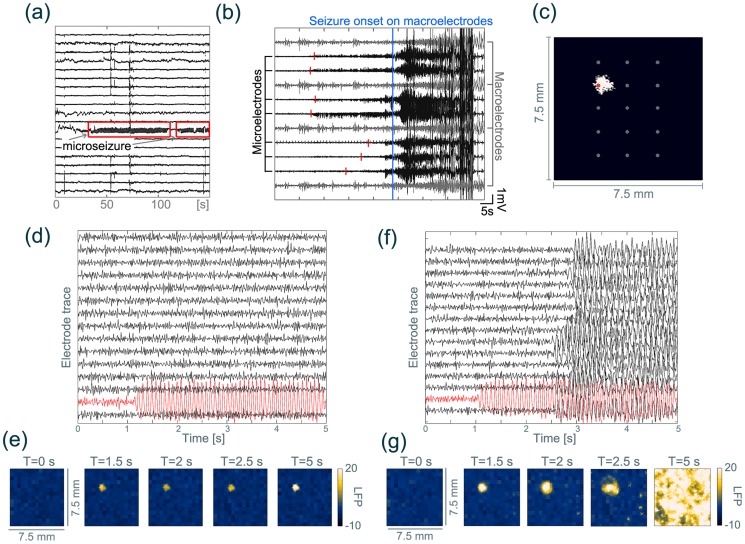
Oscillatory microdomain embedded in monostable and bistable surroundings. (**a**) Clinical microseizure recorded from microelectrodes (marked in red), which stays spatially isolated. (**b**) Clinical seizure onset from microelectrodes. Seizure activity builds up in microelectrodes (red markers) before it spreads to/recruits a larger area. (**c**) Model microdomain position that has been used (white) embedded in healthy surroundings (black). Grey dots indicate microelectrode positions used in (d) and (f). Red dot marks the electrode for the red trace in (d) and (f). (**d**) Simulated microseizure that does not recruit surrounding tissue. The LFP is plotted for units as marked in (c). The surrounding system is monostable in the background setting. (**e**) Corresponding snapshots of the LFP in time for the time series in (d). (**f**) Simulated build-up of a seizure from the hyperactive microdomain. The LFP is plotted for units as marked in (c). The surrounding system is in the bistable setting (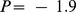). (**g**) Corresponding snapshots of LFP in time for the time series in (f). Images in (a) and (b) are data visualised from [4]. A video of the temporal evolution of the sheet for (e) and (g) is shown in Video S3 & S4. Parameter 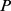 was ramped in the microdomain to the oscillatory state between 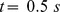 and 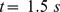. Parameter details are described in Text S5.

A perturbation to the model sheet need not be externally generated, but can arise due to local, abnormal activity generated within the model. In Fig. 6 we demonstrate that the existence of an oscillating patch in the sheet can also trigger a transition into seizure dynamics.

Fig. 6 (d, e) demonstrate that if the parameter setting of the surrounding sheet is monostable in the background state, the hyperactive microdomain remains isolated in its epileptic activity (red trace in Fig. 6 (d)). This agrees with the clinical observation of spontaneous microseizures that remain spatially localised and do not recruit surrounding tissue. If, however, the rest of the system is in a bistable setting, a continued local perturbation by an oscillatory microdomain can start to recruit the surrounding units into the seizure state (Fig. 6 (f, g)).

The propagation pattern of recruitment is similar to the case of recruitment following a pulse stimulation to the bistable sheet. Depending on the connectivity settings, smooth propagating waves, patchy propagations, or a mixture of both can be observed. Text S7B demonstrates that an oscillatory microdomain can produce recruitment speeds of between 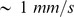 and 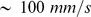, using some example parameter changes in 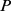. This is within a range of propagation speeds reported experimentally (0.1–100 m/s [63]).

In order to check the robustness of this onset mechanism, we tested the dependency of recruitment on both the parameter setting of the surrounding and the size of the pathological microdomain (see Fig. S12 and Text S8A). We find that for a fixed bistable parameter setting, a minimum threshold exists for the number of units that are required to induce recruitment. When the parameter setting lies closer to the monostable oscillatory setting, fewer units are required for recruitment. This behaviour is stable with different noise inputs and microdomain positions in the model sheet. This finding is important for the clinical determination of pathological vs. neutral microdomains in a patient-specific context.

#### Dynamical onset mechanism

In this section we have introduced a category where seizures are supported by a global bistability condition and initiated by a local hyperactive microdomain. We refer to it as *globally supported focal-onset seizures triggered by local oscillations*. This class is very similar to class IIa in many aspects. However, technically, the transition in this class is not a change of dynamics in a bistable system but a bifurcation induced by the (local) parameter change. The parameter crosses a critical value beyond which mono-stable autonomous oscillations are found. Globally, the background state looses stability in the presence of a persistent oscillatory drive.

#### Nature of the focus

The onset of the seizure rhythm is at the location of the trigger that induces the transition to the seizure state. In the clinical setting, this could be a local area displaying abnormal activity over a prolonged period, e.g. detectable as focal interictal activity or microseizures.

#### Distinguishing features and propagation pattern

The main feature is a discoverable local abnormal activity and a bistable surrounding. The propagation dynamics are as in class IIa.

#### Clinical relevance & predicted treatment

The points in class IIa that we already mentioned still hold in this class. Additionally, the parameter changes that drive the surrounding to become bistable, and the parameter changes that create the abnormal microdomain can be different processes clinically, with very different causes. Separately, neither might be pathological [4], [8]. Only the co-occurrence of the two processes leads to seizure onset and evolution. In patients, prevention of either process could prevent seizures. A single hyperactive microdomain can be surgically removed. In addition, there is the possibility to destroy its integrity by, for instance, cutting its connections in a microdomain using microincisions. This idea is illustrated in the Text S9. It is further supported by [64].

As the transition is caused dynamically by a bifurcation (in contrast to class IIa), local temporal pulses will not be able to fully recover the normal background rhythm for a prolonged time.

### Class III: Network induced focal seizure

After demonstrating focal seizure onset in a globally oscillatory and a globally bistable scenario, we now turn to the case of a globally monostable background. We shall investigate a system in the monostable background state except for one or multiple localised hyperactive microdomains. We examine the spatial conditions under which these hyperactive patches can recruit their surrounding, even though globally the oscillatory state neither exists exclusively (class I) nor coexists (class II) in the absence of these patches.

### Class IIIa: Autonomously oscillating network

**Figure 7:**
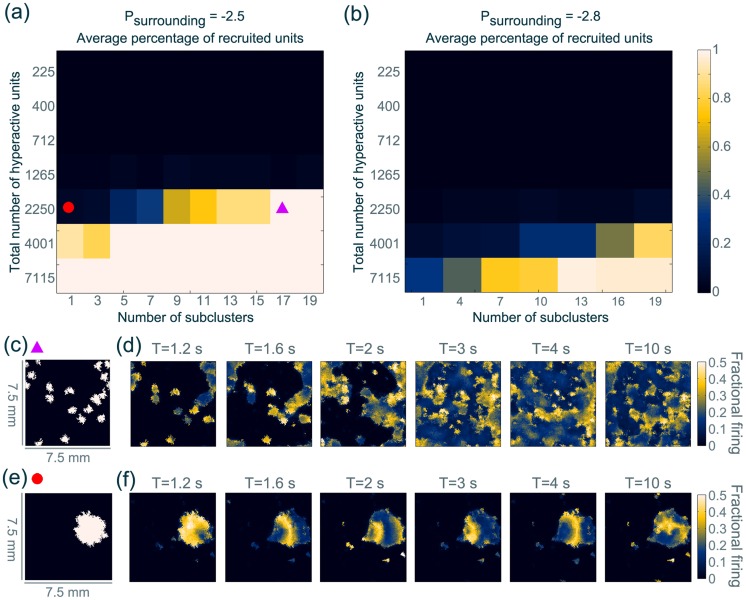
Networks of oscillatory microdomains can recruit the monostable background surrounding. (**a**) Scan of recruitment depending on the total number of hyperactive units and the number of subclusters they form on the sheet. The surrounding was set in the monostable state 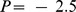. (**b**) Same scan as in (a), only using 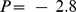 for the surrounding. (**c**) Position of the 15 sub-clusters of hyperactive units used for (d). (**d**) Snapshots of the sheet dynamics (fractional firing activity of the 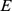 populations). The parameter 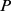 in the microdomain is ramped from the standard value of the surrounding (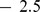) to 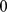 between 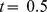 and 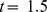. (**e**) Position of a single cluster of hyperactive units as used in (f). (**f**) Snapshots of the sheet dynamics (fractional firing activity of the 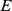 populations). Otherwise the simulation followed the same protocol as (d). Videos of the temporal evolution is shown in Video S5 and S6. Parameter details are described in Text S5.

We prepare the system in the monostable background state (the standard interictal parameter set), except for some oscillatory microdomains. We begin by systematically assessing how the recruitment from these microdomains depends upon the total number of hyperactive units and the number of subclusters that microdomains are organised into. Here a subcluster is a contiguous patch of units, positioned randomly on the sheet. Fig. 7 (a) shows that despite the surrounding being in the monostable background state, partial or full recruitment can be registered in some configurations. E.g. Fig. 7 (a) demonstrates that when 2250 units (10% of the whole sheet) are hyperactive, no recruitment is registered if all the units are organised into one compact patch (red dot). However if this same number of hyperactive units are organised into 17 subclusters of equal size, randomly distributed over the model sheet, noticeable recruitment can be observed (purple triangle). The recruitment behaviour additionally depends on the exact parameter of the surrounding. For example if the exogenous input parameter, 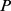, is set to a value closer to the global bistability (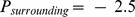, Fig. 7 (a)) recruitment starts at a lower total number of hyperactive units and with a lower number of subclusters than when using 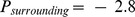, further away from the bistability (Fig. 7 (b)). This recruitment behaviour is stable with regards to the noise input in our simulation. However, the exact values of the total number of hyperactive units and the number of clusters vary slightly with the position of the (sub)clusters (see Fig. S13, Text S8A).

Fig. 7 (d,f) show example time series from two simulations using the same number of hyperactive units (2250 units, 10% of the whole sheet), but different numbers of sub-clusters. In the case of a single cluster, only a few units are recruited (Fig. 7 (f)). In the case of many sub-clusters, the whole sheet is recruited (Fig. 7 (d)). Recruitment can be observed to begin in areas of increased subcluster density (for example right side of T = 1.6 s in Fig. 7 (d)). The “normal” monostable tissue between nearby subclusters is recruited first. In this way, the subclusters that lie in close proximity recruit the healthy tissue between them to form a bigger contiguous cluster of oscillatory activity (T = 2 s in Fig. 7 (d)), and eventually recruit the whole cortical sheet (T = 4 s, T = 10 s in Fig. 7 (d)).

The recruitment of monostable surrounding tissue in the background state is not as intuitively understandable as for instance the case of recruitment of a bistable surround. The scans in Fig. 7 (a,b) show that the spatial arrangement of “recruiters” is important. We propose that the basic mechanism is based on the coherent oscillatory input to units in the background state, which can incite them to oscillate despite their configuration being monostable. The parameter change in the microdomains induces the microdomains to become intrinsically oscillatory. Hence, the recruitment from microdomains induced by this local parameter change is a bifurcation from a node to an oscillatory state. The onset of oscillations, while ramping 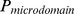, occurs with a sudden change in frequency and amplitude. We additionally address the effect of boundary conditions on this mechanism in Text S2.

#### Dynamical onset mechanism

Appearance of autonomously oscillating patches on a monostable cortical sheet induces a system-wide bifurcation to the oscillatory state. We refer to this class of onset mechanism as *active microdomain network induced focal seizures*.

#### Nature of the focus

The focus consists of an assembly of autonomously oscillating tissue patches. Patches that are in close proximity to each other can recruit tissue between them from a monostable background to an oscillatory state. This way, larger patches form, which in turn are able to recruit tissue between them. The recruitment eventually leads to formation of a growing patch of autonomously oscillating tissue. The growth/recruitment is always between seed patches, which display autonomous oscillations.

#### Distinguishing features and propagation pattern

A clear distinguishing feature of this class is the presence of hyperactive microdomains. Clinical cases in which microscopic localised seizure-like activity is registered, and which develop into a full-recruitment seizures, are likely to belong in this class. Single pulse stimuli would not induce recruitment as only the autonomous onset of oscillations in microdomains determines if and when recruitment starts.

The propagation pattern of recruitment is from one active microdomain to the next, depending on the position of the microdomains relative to the underlying connectivity. Multiple “foci” could be observed, starting from several dense hyperactive microdomain regions. These foci would then coalesce to form a larger continuously recruited area, which might then form for instance the ictal symptomatogenic zone by the definition of [1]. In the scenario of a strongly connected remote excitation network and weakly connected local network, the propagation pattern could be saltatory, advancing from patch to patch, as shown in the previous class.

#### Clinical relevance

Micro-seizures, or micro-periodic discharges have been recorded on the human cortex by [4], [5], [65] and could represent examples of hyperactive localised domains. Increased occurrence rates, as well as increased spatial density of these micro-seizures have been correlated with the seizure onset zone. Our model observations are in line with those findings. If the occurrence rates of micro-seizures can be registered for a part of the cortex around the seizure onset zone (using e.g. high-density microelectrode arrays), an increased occurrence rate of abnormal microactivity in a susceptible area could be used as an estimator for the likelihood of an impending seizure.

#### Predicted treatment

The drivers of the transition to the seizure state are the hyperactive microdomains. Hence treatment can aim to prevent the increased occurrence of microseizures by identifying and targeting the generating mechanisms. Alternatively, a global parameter change towards decreasing global excitation can also help to increase the threshold for the number of required hyperactive domains to elicit recruitment (compare Fig. 7 (a) and (b)). This effect can potentially explain why treatment using anti-epiletic drugs (AEDs) is successful in some patients with partial seizures. However this does not remove the cause of the seizure onset and patients might still be susceptible to some global and local parameter variations, which are not controlled by the AEDs.

Similar to the class II mechanism, in terms of surgical interventions, destruction of the integrity of hyperactive microdomains (effectively reducing their size) would prevent recruitment. Alternatively the connections between microdomains and their surrounding could be disrupted to prevent recruitment.

The mechanism of micro-seizure appearance should be further investigated and patient-specific connectivity of the microdomains and surrounding local tissue should be assessed to determine the optimal intervention method.

### Class IIIb: Bistable network

We have shown that clusters of autonomous oscillations can induce recruitment of the whole system to the seizure state. In this section we investigate additionally whether a system-wide bistability can be induced by localised, bistable clusters of tissue (i.e. a set of bistable microdomains). The reasoning is that if the network of microdomains is bistable, specific localised stimuli will be able to induce localised oscillatory behaviour in the patches, which in turn would lead to recruitment of the monostable surrounding as in class IIIa.

**Figure 8:**
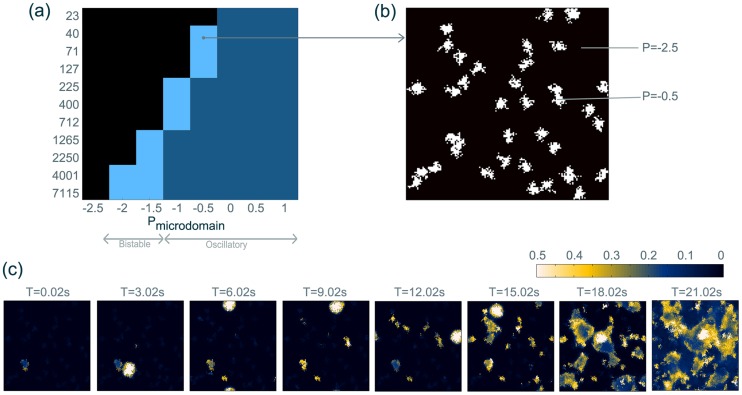
Bistable microdomains can recruit monostable background surrounding. (**a**) Microdomain behaviour depending on microdomain size and its basal excitatory input (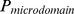). 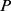 of the surrounding is in this case monostable. Dark (light) blue indicates parameter regions, where the microdomain is monostable oscillatory (bistable). The grey arrows on the bottom indicate the corresponding parameter regions for global parameter variations (derived from Fig. 3). (**b**) Position of the bistable microdomains used in (c). (**c**) Temporal snapshots of an example time course, where a sheet with bistable microdomains is perturbed at T = 0,3,6,9,12,15,18 and 21 s with local, arbitrarily placed stimuli. A video of this simulation is shown in Video S7 Parameter details are described in Text S5.

For such a scenario, it is required to determine the conditions under which a local cluster of tissue is bistable. Hence we scan the size of a microdomain embedded in a monostable background surrounding versus an exemplary local parameter change (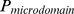) and determine whether a microdomain patch is bistable by applying a single-pulse stimulus (Fig. 8 (a)). An elevation in 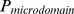 leads to bistability of the microdomain. Upon further increase of 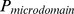, the microdomain becomes monostable oscillatory. This bifurcation also occurs with a sudden change in amplitude and frequency. As the patches become smaller, 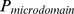 has to be higher to reach bistability (or the monostable oscillations) in the microdomain. The dependency of the dynamic behaviour on the size of the microdomain can be understood if we consider that the oscillatory state in the system emerges from the coupling of individual units.

Using information from the previous parameter scan, we set up a monostable sheet and distribute bistable microdomains within it (Fig. 8 (b)). Such a system remains in the monostable background state in the absence of perturbations. Multiple single-pulse stimuli applied randomly at different locations can be used to activate some bistable patches (Fig. 8 (c)). Some degree of coactivation (i.e. an active patch subsequently activating a connected silent patch) can also be observed. Once activated and in high enough density, the patches can cause recruitment of their non-oscillatory environment as shown in the previous section. Fig. 8 (c) shows a time course of multiple stimuli activating silent bistable patches, which ultimately results in full recruitment of the sheet.

#### Dynamical onset mechanism

In this class, the recruitment is a combined effect of some tissue heterogeneity (which is not observable without perturbations) and some (not necessarily pathological) stimuli. Hence, we term this class *microdomain network supported focal seizures*. Dynamically, the bistable microdomains introduce a bistability, where targeted perturbations can induce a transition of the whole sheet to the oscillatory state.

#### Nature of the focus

Initially, stimuli only activate isolated bistable patches. These patches can stay activated for a long time without recruiting their neighbourhood. The seizure recruitment requires the co-activation of connected microdomains through successive stimuli. After the co-activation, the recruitment progresses as in the previous class.

#### Distinguishing features and propagation pattern

The propagation pattern in this class is very similar to the previous class IIIa. The difference is that recruitment only progresses between activated patches. Silent patches do not aid recruitment, except when connected neighbours are in the seizure state. Active stimulation could be able to highlight the location of silent microdomains clinically, when tested prior to seizure events.

#### Clinical relevance

This class has implications for clinical observations of responses of epileptic tissue to stimulation. The effect of abnormal, localised activation of epileptic tissue following stimulation is well known as after-discharges [66]. The clinically observed after-discharges could be compatible with the activation of subnetworks of bistable patches, i.e. the subset of bistable patches activated by continued stimulation without recruitment of the monostable surrounding into a full seizure. This hypothesis could be tested systematically in experimental models. Clinically, it would be important to record the spatio-temporal characteristics of epileptiform afterdischarges with high spatial resolution (e.g. during presurgical monitoring).

#### Predicted treatment

Similar to the previous class, destruction of the integrity of the (silent) microdomains would prevent full recruitment, as would disconnection of these microdomains. However, extensive single-pulse stimulation in a setting with high spatial resolution would be required to identify the locations of the abnormal microdomains.

As in class IIIa, a global parameter change (towards less excitation for each unit) can increase the threshold for the number of required hyperactive domains to elicit recruitment. An additional possibility in the current class of onset mechanism is to prevent or counteract the seizure-inducing stimuli.

Finally, we summarise the most important point in all three classes in Table 1.

**Table 1:** Summary of the three classes of onset mechanisms.

Class	Global setting	Spatial heterogeneity	Stimulation	Related clinical observations	Possible treatment
I	Oscillatory	Silent interictally, appears as seizure focus at onset	-	Increased global excitability of the cortex before seizure onset [6], [8], [9]; Secondary foci [1]	Global acting drugs; Identify cause of increased global excitability.
IIa	Bistable	-	Local temporary stimulus	Peri-ictal state [6], [8]; Reflex epilepsies	Global acting drugs; Identify cause of bistability; Prevent stimulus; Counter stimulation.
IIb	Bistable	Oscillatory microdomain	-	Peri-ictal state [6], [8]; Microseizures [4]	Global acting drugs; Identify cause of bistability; Remove microdomain; Microincisions.
IIIa	Monostable background	Oscillatory microdomains	-	Microseizures [4], [5], [65]	Global acting drugs to increase seizure threshold; Remove microdomains; Microincisions through the microdomain or between microdomains.
IIIb	Monostable background	Bistable microdomains	Multiple stimuli	Microseizures [4], [5], [65]; After-discharges [66]	Global acting drugs to increase seizure threshold; Remove microdomains; Microincisions; Counter stimuli.

## Discussion

In this study we used a novel spatio-temporal model of the dynamics of cortical minicolumns, coupled by multi-scale cortical connectivity, to categorise possible mechanisms of focal seizure onset. We showed that in this framework, apparently conflicting clinical observations regarding focal seizure onset can be understood and unified. We furthermore suggested how to test for the different onset categories, and made predictions regarding possible treatment methods for each category.

The three mechanisms we identified by which a focal seizure onset can occur are: (I) A global parameter change which induces a global bifurcation of a piece of cortical tissue to the seizure state. (II) A global bistability combined with a local trigger leading to transition to the seizure state. (III) A globally monostable state with local parameter changes causing recruitment of the whole system. We expect that either mechanism may dominate the onset of focal seizures in different patients.

The model employed herein uses the approach of discretised, coupled spatial units to reflect the activity of a piece of contiguous cortical tissue. Each unit in the current model is described by Wilson-Cowan equations, which embody the collective activity of local excitatory and inhibitory neural populations [40]. Compared to detailed neuronal models of cortical activity (e.g. [67]), the Wilson-Cowan model is computationally less demanding and the number of parameters to analyse is manageable. However the parameters of the Wilson-Cowan model are more abstract in nature. Thus, if for example cellular mechanisms of focal seizure onset are to be investigated (e.g. [68]), a detailed neuronal model is required. Similarly, if the detailed laminar and horizontal interaction between different types of excitatory and inhibitory populations is of interest, the populations in our model can be extended. However, in our current study, describing the dynamics of cortical minicolumns in terms of the lumped activity of generic excitatory and inhibitory neural populations allowed us to model a hierarchy of clinically relevant spatial scales by reducing the level of detail for the analysis.

The classical Wilson-Cowan model has been used to reflect EEG/ECoG dynamics in the delta to beta range [41], [69]. Similarly, we used it here to model seizure oscillations in this frequency range. Faster or slower dynamics are therefore not considered in our current approach, although it will be interesting in future studies to investigate the influence of these aspects, for example the addition of slower time scales. The incorporation of additional intrinsic long-term dynamics (e.g. adaptation or learning) can lead to the creation of additional types of dynamics, which could also be relevant for clinical question. If the time scale separation is sufficient (i.e. intrinsic long-term dynamics are on the order of seconds or longer) Fenichels theorem [70] indicates that our presented attractors would remain as manifolds in the full system with a slower time scale. Hence the slower time scale dynamics would modulate and orchestrate the transitions between the stable dynamics presented here. Indeed, the global parameter configuration (monostable, bistable, and oscillatory) used in our current model could be fluctuating over time according to some slow dynamics. It might be that the parameters of the cortex of patients as well as healthy subjects are constantly changing [8], putting cortical tissue in different global configurations at different times. However, in an epileptic patient, either these global fluctuation are either too extreme leading to a global bifurcation into the seizure state (class I), or would remain silent if not co-occuring with a local trigger (class II), or do not affect seizure onset directly (class III). In patients with stereotypical seizure onset (i.e. the seizure onset is repeatedly from the same region with a similar electrographic pattern), the underlying long time-scale dynamics are either similar from seizure to seizure, or at least giving similar dynamical conditions. Hence the categories would apply to all seizures of the same stereotype (in the same patient). Our classification is hence crucial to determine (patient-specifically) the exact role of the parameter fluctuation dynamics in seizure onset. Practically, a constant multi-scale monitoring of the cortical activity, as well as regular stimulation tests should be carried out to determine the global and local parameter configuration.

In our model, we equated high amplitude oscillations with a pathological state in each mini column. This is mainly inspired by the observation that the seizure core contains highly active neurons with firing patterns phase locked to the oscillatory LFP [54]. We believe that in our case, firing activity might provide a better benchmark for comparison of clinical and simulation data than LFP, as the generators of the different components of focal seizure field potentials are largely unknown. Hence, following [54], we identified high amplitude oscillations in firing as the seizure state and low level firing as the background state. Additionally, the approach of identifying oscillations with seizures and fixed points with background activity is well established in the modelling literature (see for example [51], [55], [56], [71], [72]). It is in line with the long-standing suggestion of dynamic diseases [73], [74], where the disease state is identified as an oscillatory attractor and the background state as an non-oscillatory, primarily noise dominated state. Only very little clinical or theoretical understanding exists regarding the different waveform morphologies in focal seizures [75], [76] and how seizure onset mechanisms influence them. Weiss *et al*. (2013) [77] point out that high frequency oscillations phase locked to low frequency oscillations at seizure onset could be an indicator for increased, structured firing in the underlying tissue and hence an indicator for the seizure core. Future studies should specifically investigate how focal seizure onset field potential morphologies arise, as well as how they relate to firing patterns. Potentially, the knowledge gained by studies of waveform morphology in purely temporal framework such as [41], [71], [78] could be of use.

Each of the onset mechanisms we describe relies on a certain configuration of global parameters, where global is in reference to the scale of the model of about one square centimetre of cortex. However, in reality global parameter changes in the brain will vary from the whole-brain level to the scale of our current model, all of which can influence the global parameter configuration in our model. A range of physiological and pathological conditions could cause such variations. For example different phases of the sleep-wake cycle or hormonal variations [8] can change the excitability of brain. Pathological conditions include misregulation of excitation and inhibition [10]. If pathological parameters changes exist in a limited part of the cortex, then the focal seizure could be limited in its spatial extent. However, if abnormal dynamics entrain a large region, they could activate other whole-brain networks (including subcortical networks) leading to generalised abnormal activity (secondary generalisation).

In this context the model can also resolve the apparent contradictions in the experimental literature on the mechanisms of focal seizure onset. The contradiction of focal seizure onset being a result of global (whole brain network changes) or local (abnormally behaving cortical columns) mechanisms is no longer a contradiction in our model. We have shown that global as well as local mechanisms can interact and we have classified the interaction in three major categories. Hence, global changes can cause (class I), or support (class II), or modify (class III) seizure onset. Equally, local changes can trigger (class II), cause (class IIIa), or support (class IIIb) focal seizures. It is hence no surprise that clinical and experimental observations supporting both global as well as local mechanisms are found. Similarly, the contrasting observations from [11] and [54] can also be united: it might be that very near to the “focus” recruitment propagates as a wave over the local network. However, further away regions are probably recruited via remote or long-distance connections first and activation is primarily patchy. Hence, the conflicting recruitment dynamics described by Truccolo *et al*. (2011) and Schevon *et al*. (2012) is explained in our model by the propagation of activity via different networks. Interestingly, Schevon *et al*. (2012) [54] hypothesised that the ictal penumbra could restrain the propagation of epileptic activity due to an “inhibitory veto”. In our model of non-recruiting microdomains, we find that the restraint is not explicitly excessive inhibitory firing activity in the penumbra. Rather, the net synaptic input into each unit in the penumbra is not strong enough to entrain them to become oscillatory.

A question that arises from our study is whether the categories we established can be generalised to any spatio-temporal system showing bifurcations or bistabilities between a non-oscillatory (fixed point) and an oscillatory state. We propose that the detailed transition dynamics will depend on the specific system. However, we postulate that the three categories are general features of spatio-temporal systems showing either a bifurcation or a bistability between fixed point and oscillation. This is mainly due to the observation of the three categories in other spatio-temporal models using different model formalisms as well as underlying connectivity. For instance [17] essentially show class IIa in their partial differential equation model. Class I has been shown in a coupled Amari-type model representing a whole-brain network [24]. [64] show a class IIIa transition in their rule-based model of microseizures and recruitment. To our knowledge, class IIIb has not been demonstrated explicitly so far. We emphasise that the dynamical classification only becomes useful in the context of a relevant model, and the interpretation becomes useful when it is applied to the clinical context, e.g. to search for the cause of the seizure and to devise potential treatment strategies.

We have outlined major features and the expected observations of each class of onset mechanism in the Results section. A question that remains is how one would practically tell the classes and subclasses apart in a clinical setting. This question is crucial, as treatment will depend on the individual mechanism of seizure onset in a patient. We suggest that high resolution spatio-temporal recordings, similar to [79], combined with local perturbation studies (similar to [80], but on different spatial scales) might be the key to answer this question. In the context of local perturbations, we point out that although we only demonstrated the impact of pulse stimulation in our current study (to essentially reset the activity of the excitatory population), practically the effect of different types of stimulation has to be assessed prior to its usage for the classification of the seizure onset.

In this context, we recommend the development of patient-specific models to classify the dynamic seizure onset mechanism. This would involve incorporating the patient-specific connectivity of the affected cortical area (e.g elucidated from high resolution track density imaging [81]–[83]), as well as online parameter fitting according to passive and active high-resolution spatio-temporal recordings. This could enable the use of closed loop counter-stimulation devices (as demonstrated in Fig. S15). Additionally, such patient-specific models can be employed to predict optimal treatment protocols, for example minimal cortical micro-incisions to stop the recruitment of tissue into full seizures (see Fig. S16 and [84]).

In the case of a global shift of parameters (affecting larger brain regions) causing or facilitating seizure initiation and recruitment, it is probably desirable to target the reason for the global shift directly rather than trying to suppress seizure onset locally. In fact class I onset demonstrates that although one particular cortical location appears to be the source of seizure initiation (epileptogenic zone), the mechanism causing the seizure can be a global parameter shift in an extended tissue. The “epileptogenic zone” only reacts first due to its increased local threshold. Then, despite reducing or removing the local activity in the seizure onset zone, the seizure still starts, albeit from a different “most active” site. This concept of the existence of alternative foci has been proposed from clinical reasoning [1], [3] to explain why some surgical resections of epileptogenic zones have little effect.

Conceptually, we hence propose to distinguish between global or generalised causes of focal seizures, which induce the seizure by a global parameter shift - and local or focal causes of seizure, which can be facilitated by global bistability settings. The spatial extent of the cause of the seizure, however, can differ greatly from the spatial extent of the observed seizure onset. The traditional concepts of the epileptogenic zone and the seizure onset zone do not fully account for this. The understanding and treatment, of focal-onset seizures might benefit from further clinical and computational studies of seizure onset mechanisms on multiple spatial scales.

## Supporting Information

Figure S1 **Degree and distance distribution of the connectivity in our model.** (a–c) In degree distribution of the three connectivities. (d–f) Out degree distribution of the three connectivities. (g–i) Distance distributions of the three connectivities. Y-axis always indicates the count number.
(TIF)

Figure S2
**Recruitment on a monostable sheet using zero-flux boundary conditions.** In this scan 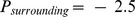 was set in the monostable state. All simulations and scans performed on a system with zero-flux boundaries (ZFB). This figure is the equivalent of Fig. 7 using ZFB. (**a**) Scan of average percentage of recruitment with respect to the total number of hyperactive units and the number of subclusters these are grouped in. (**b**) Location of the hyperactive units for (c) and (d), respectively. (**c**) Snapshots in time using one cluster of hyperactive units. Minimal recruitment (4.4%) can be observed. (**d**) Snapshots in time using 25 clusters of hyperactive units. Recruitment (70%) can be observed for regions between the clusters.(TIF)

Figure S3
**Oscillation amplitude of a single E-I unit.** Oscillation amplitude is indicated as a colour-code for different values of 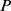 and 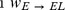. Black indicates the background fixed point. The grey region additionally shows the parameter region for where an upper fixed point exists. The current setting of the single unit is indicated with the red dot.(TIF)

Figure S4
**Single E-I unit phase space.** 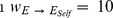 for all plots. Increases in 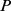 changes the lower fixed point node to a focus (via a saddle-focus and a saddle-node bifurcation) between 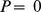 and 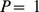. No limit cycles are found with increasing 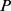.(TIF)

Figure S5 **Bifurcation behaviour of the full system in self excitation and inhibition.** Black indicates monostable background. Light blue indicates bistable oscillatory state and background state. Striped yellow indicates bistable background and upper fixed point. Red dot marks the interictal standard parameter position used throughout the manuscript.
(TIF)

Figure S6
**Input pulse vs. initial condition reset pulse.** 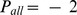 has been used for all simulations, putting the sheet in the bistable regime. (**a**) Stimulus position on the simulated cortical sheet. This is used for both types of stimuli. (**b**) The input pulse used to simulate input stimulation (c) and (d). (**c**) Time series of the average macrocolumn excitatory populations for an input pulse given at T = 1 s. (**d**) Corresponding snapshots in time for (c). (**e**) Time series of the average macrocolumn excitatory populations for an initial condition reset to 1 at T = 1 s. (**f**) Corresponding snapshots in time for (e).(TIF)

Figure S7
**Simulating the changing motor threshold in the lead-up to a seizure.** The seizure (see (a,b) for time series) has been induced using a global parameter ramp in 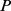
(c). The measured motor threshold in the model is shown in (d).(TIF)

Figure S8 **Example propagation patterns using local or remote connections.** Snapshot of the sheet at different time points for strong local connection weights, weak remote connection weights (top), and strong remote connection weights and weak local connection weight (bottom). A mixture between both dynamics can be seen in Fig. 5 (d) of the main manuscript.
(TIF)

Figure S9 **Recruitment dynamics for different local and remote feed-forward connection weights.** (a) Number of recruited units 5 s after stimulus, depending on the remote and local connection strength. (b) Time required to recruit half of the final recruitment number as shown in (a).
(TIF)

Figure S10
**Percentage of recruitment over time and different conditions of the surrounding.** (**a**) For different values of 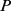, recruitment (colour code) following a single pulse stimulus was measured every 0.3 s. (**b**) For different values of 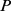, recruitment (colour code) from a hyperactive microdomain was measured every 0.3 s. (**c**) For different values of 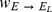, recruitment (colour code) following a single pulse stimulus was measured every 0.3 s. (**d**) For different values of 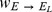, recruitment (colour code) from a hyperactive microdomain was measured every 0.3 s. The stimulus location and the location of the hyperactive microdomain were identical in all scans. Each scan point is obtained as the average over 5 different noise inputs.(TIF)

Figure S11
**Recruitment depending on** 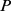 **and number of units being stimulated.** In this scan the stimulus was to contiguous patches on the sheet. (a) Scan result obtained by averaging over different noise inputs, 5 for each of the 5 stimulation positions. The 5 positions were chosen at random. Colour code indicates percentage of recruited units relative to the total number of units. 1 on the colour bar indicated 100% recruitment. (b) The maximum difference in terms of recruitment between different microdomain positions of the same setting (averaged over 5 different noise inputs for each position). (c) The maximum difference in terms of recruitment of the surrounding between the 25 different noise inputs of the same setting, off-setted against the already registered effect of the microdomain position.(TIF)

Figure S12
**Recruitment depending on** 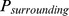 **and number of units in the hyperactive microdomain.** In this scan only one continuous microdomain was used. (a) The scan result is obtained by averaging over different noise inputs, 5 for each of the 5 microdomain positions. The 5 positions were chosen at random. Colour code indicates percentage of recruited units relative to the total number of units outside of the microdomain. 1 on the colour bar indicated 100% recruitment of the surrounding. (b) The maximum difference in terms of recruitment of the surrounding between different microdomain positions of the same setting (averaged over 5 different noise inputs for each position). (c) The maximum difference in terms of recruitment of the surrounding between the 25 different noise inputs of the same setting, off-setted against the already registered effect of the microdomain position.(TIF)

Figure S13
**Recruitment depending on the number of subclusters and number of hyperactive units.** In this scan 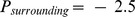 was set in the monostable background state. (a) The scan result is obtained by averaging over different noise inputs, 5 for each of the 5 microdomain positions. The 5 positions were chosen at random. Colour code indicates percentage of recruited units relative to the total number of units outside of the microdomain(s). 1 on the colour bar indicated 100% recruitment of the surrounding. (b) The maximum difference in terms of recruitment of the surrounding between different microdomain positions of the same setting (averaged over 5 different noise inputs for each position). (c) The maximum difference in terms of recruitment of the surrounding between the 25 different noise inputs of the same setting, off-setted against the already registered effect of the microdomain position.(TIF)

Figure S14
**Variation of the** 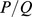 **bifurcation diagram with changing underlying connectivity.** Five different 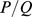 bifurcation diagrams were obtained using five different local and remote connectivities (generated by the same algorithm using the same parameters). Black indicates monostable background. Light blue indicates bistable oscillatory state and background state. Dark blue indicates monostable oscillatory state. Dotted light blue indicates a deviation in at least one of the five scans at this parameter setting.(TIF)

Figure S15
**Simulating counter stimulation.** (**a**) Snapshots of fractional firing activity of the 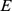 populations upon stimulation at 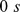. Recruitment starts from a local perturbation. (**b**) Identical system and simulation conditions as above, only with a counter-stimulus delivered at 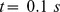 (red block). The recruitment is suppressed.(TIF)

Figure S16
**Simulating microincision.** (**a**) Snapshots from a time evolution of fractional firing activity of the 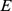 populations. Recruitment begins from a hyperactive microdomain (where its 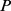 value has been ramped to 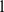 between 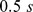 and 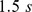). The surrounding sheet is in the bistable state. (**b**) Identical system and simulation conditions as (a), only with all connections removed that intersect the cut (red line). Recruitment is delayed and starts from only one half of the microdomain. (**c**) Identical system and simulation conditions as above, only with an additional cut (second red line). Recruitment is suppressed during the whole simulated time.(TIF)

Figure S17 **P/Q bifurcation diagram of the whole sheet for different values of the signal propagation speed.** Black indicates monostable background. Light blue indicates bistable oscillatory state and background state. Dark blue indicates monostable oscillatory state.
(TIF)

Text S1 **Additional methods and algorithms.**
(PDF)

Text S2 **Effect of boundary conditions.**
(PDF)

Text S3 **Parameter setting for the single unit.**
(PDF)

Text S4 **Additional parameter scans for the full system.**
(PDF)

Text S5 **Details of parameters and simulation methods for every figure.**
(PDF)

Text S6 **Simulating stimulation.**
(PDF)

Text S7 **Propagation patterns and recruitment speed.**
(PDF)

Text S8 **Additional parameter scans and their variability.**
(PDF)

Text S9 **Simulating seizure prevention.**
(PDF)

Text S10 **Effect of propagation delays.**
(PDF)

Video S1
**Global ramping of** 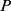 **to the oscillatory state induced seizure onset.** Same simulation conditions as Fig. 4, i.e. a small heterogeneity is introduced in the middle of the sheet. In the video we leave the system in the background state for longer by ramping 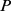 slowly. 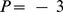 for T = 0 s until T = 0.5 s. 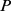 ramped from -3 to -1 between T = 0.5 s and T = 4 s. 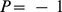 from T = 4 s until T = 5 s.(MP4)

Video S2 **Stimulus to bistable sheet induced propagating seizure activity.** Same simulation conditions as Fig. 5.
(MP4)

Video S3 **Oscillatory microdomain remains isolated in a monostable surrounding.** Same simulation conditions as Fig. 6 (d,e).
(MP4)

Video S4 **Oscillatory microdomain recruits bistable surrounding.** Same simulation conditions as Fig. 6 (f,g).
(MP4)

Video S5 **Single contiguous oscillatory microdomain remains isolated in a monostable surrounding.** Same simulation conditions as Fig. 7 (e,f).
(MP4)

Video S6 **Multiple subclusters of oscillatory microdomains recruits monostable surrounding.** Same simulation conditions as Fig. 7 (c,d).
(MP4)

Video S7 **Multiple bistable microdomains recruits monostable surrounding after multiple stimuli.** Same simulation conditions as Fig. 8 (c).
(MP4)
