# Supplementary figures and images for: Dynamic Mechanisms of Neocortical Focal Seizure Onset
Source: PLoS Comput Biol. 2014 Aug 14;10(8):e1003787. doi: 10.1371/journal.pcbi.1003787 (PMC4133160; doi:10.1371/journal.pcbi.1003787)

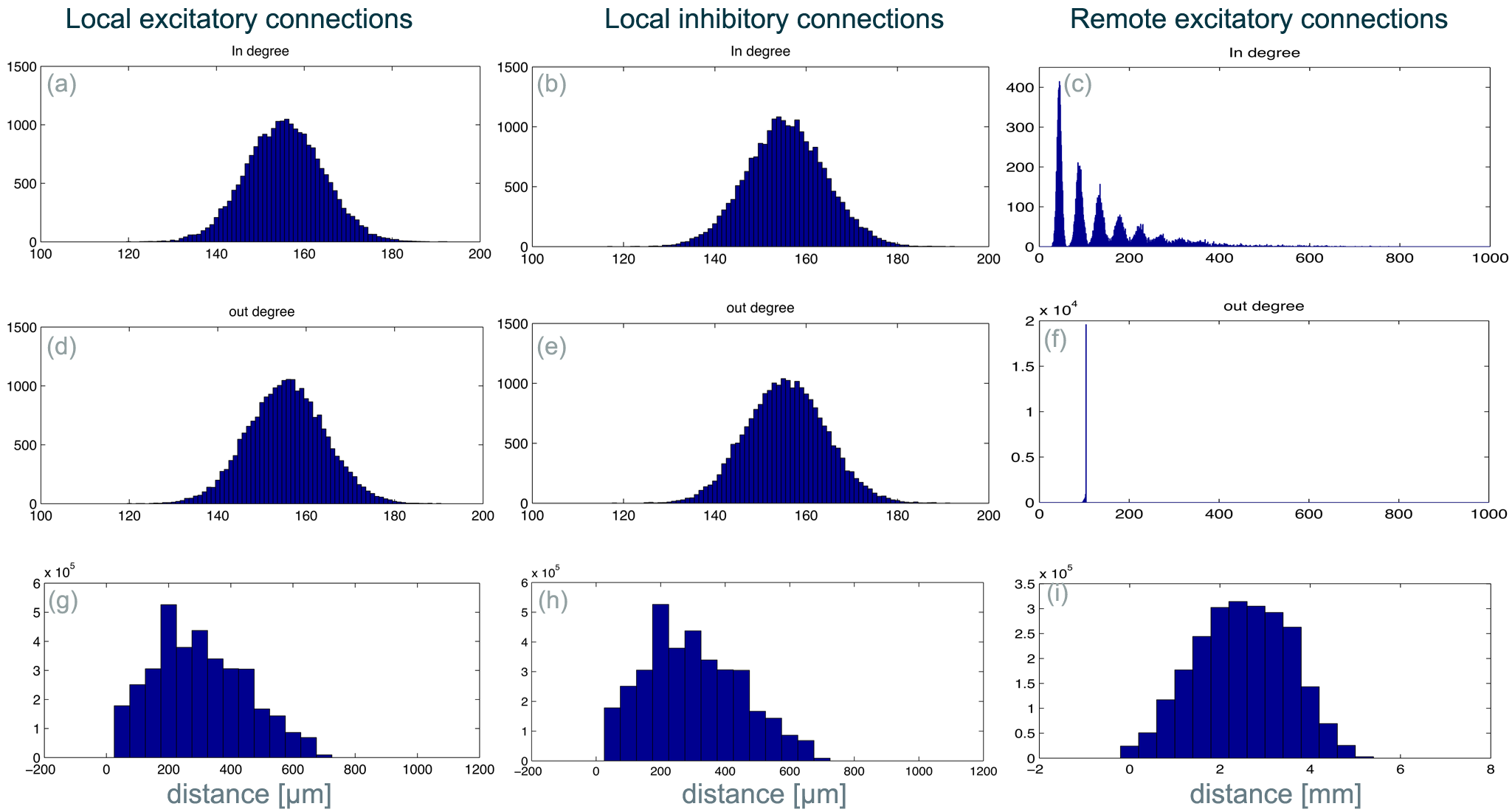

Supplement: Figure S1 — Degree and distance distribution of the connectivity in our model. (a–c) In degree distribution of the three connectivities. (d–f) Out degree distribution of the three connectivities. (g–i) Distance distributions of the three connectivities. Y-axis always indicates the count number. (TIF) [file pcbi.1003787.s001.tif]

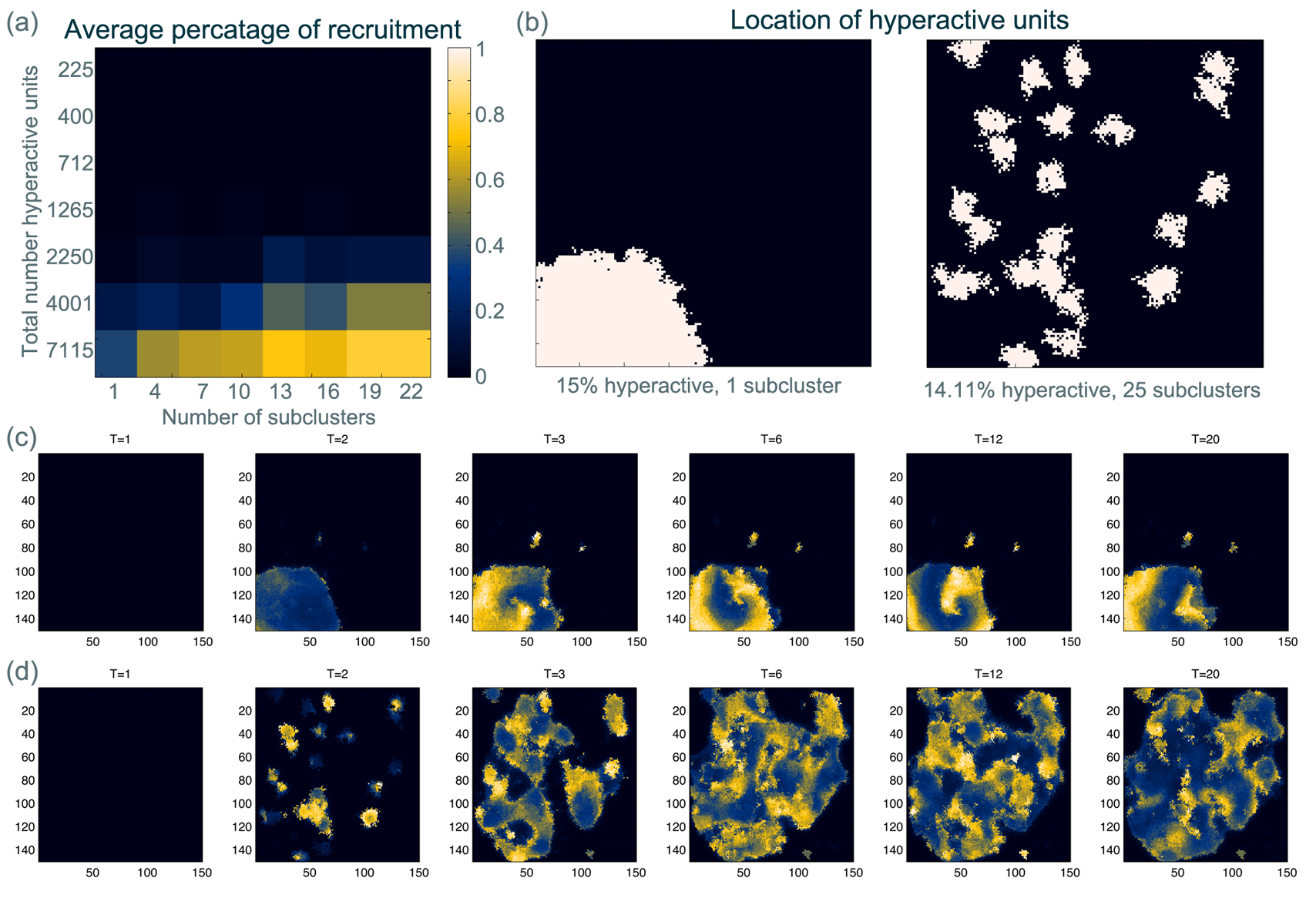

Supplement: Figure S2 — Recruitment on a monostable sheet using zero-flux boundary conditions. In this scan was set in the monostable state. All simulations and scans performed on a system with zero-flux boundaries (ZFB). This figure is the equivalent of Fig. 7 using ZFB. (a) Scan of average percentage of recruitment with respect to the total number of hyperactive units and the number of subclusters these are grouped in. (b) Location of the hyperactive units for (c) and (d), respectively. (c) Snapshots in time using one cluster of hyperactive units. Minimal recruitment (4.4%) can be observed. (d) Snapshots in time using 25 clusters of hyperactive units. Recruitment (70%) can be observed for regions between the clusters. (TIF) [file pcbi.1003787.s002.tif]

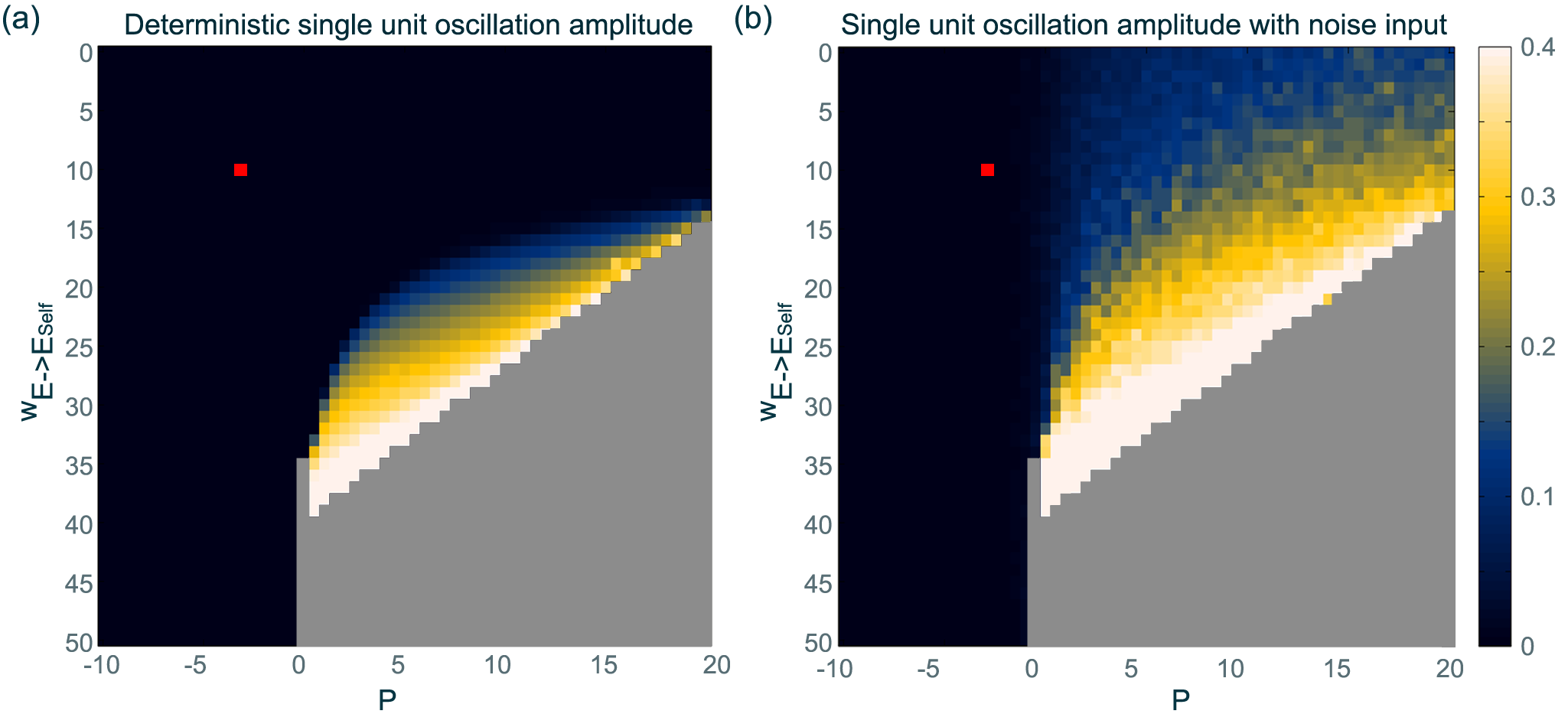

Supplement: Figure S3 — Oscillation amplitude of a single E-I unit. Oscillation amplitude is indicated as a colour-code for different values of and . Black indicates the background fixed point. The grey region additionally shows the parameter region for where an upper fixed point exists. The current setting of the single unit is indicated with the red dot. (TIF) [file pcbi.1003787.s003.tif]

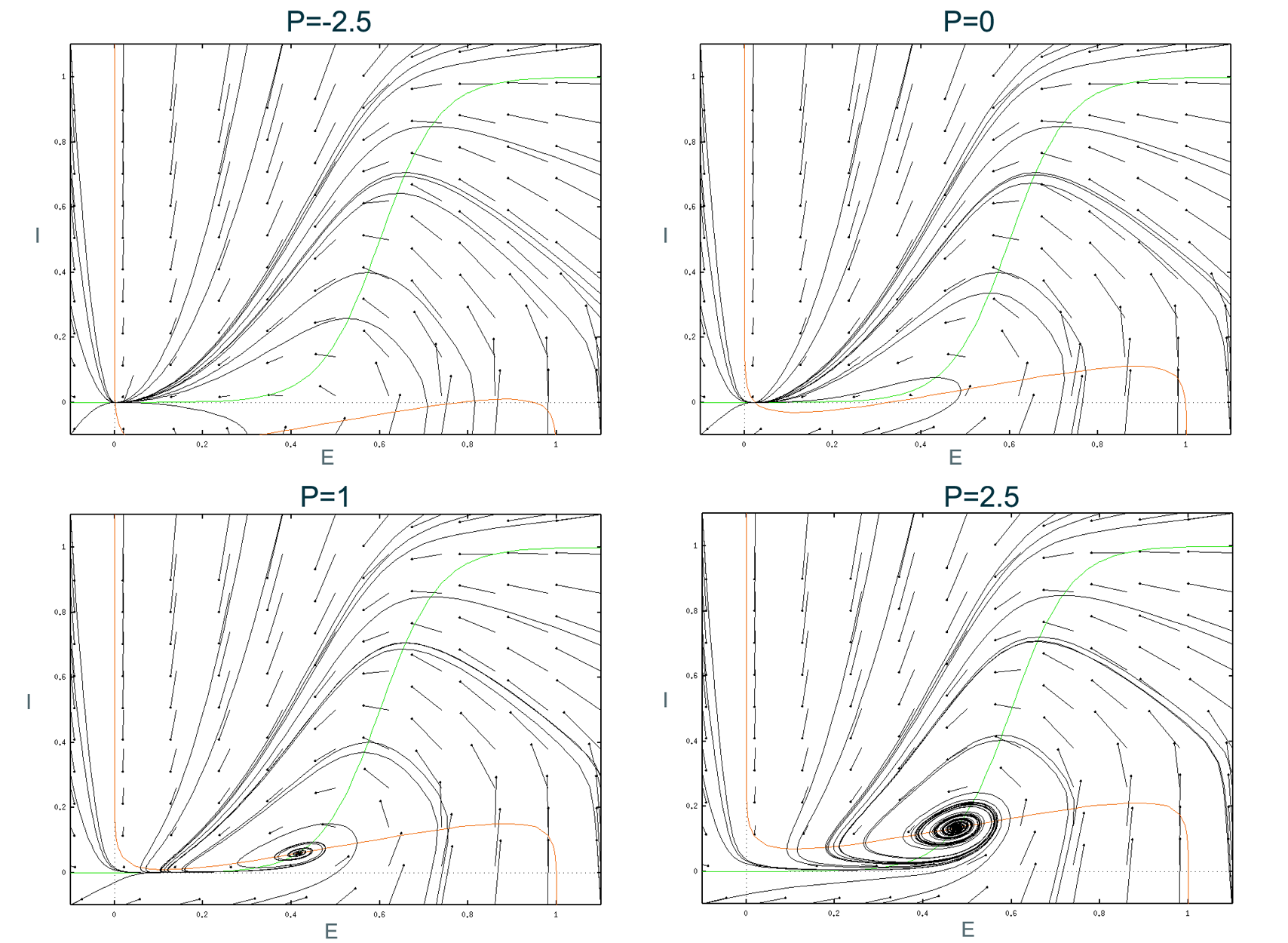

Supplement: Figure S4 — Single E-I unit phase space. for all plots. Increases in changes the lower fixed point node to a focus (via a saddle-focus and a saddle-node bifurcation) between and . No limit cycles are found with increasing . (TIF) [file pcbi.1003787.s004.tif]

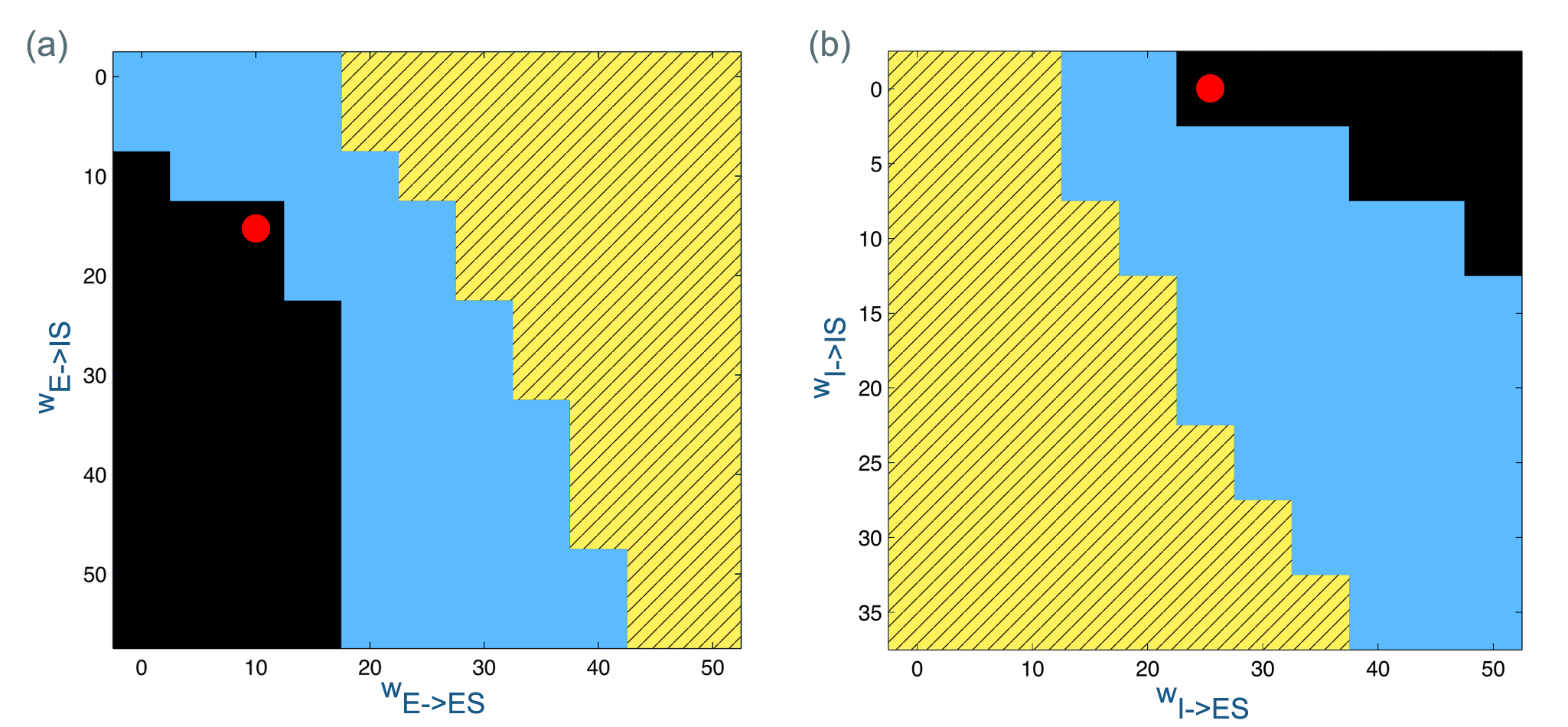

Supplement: Figure S5 — Bifurcation behaviour of the full system in self excitation and inhibition. Black indicates monostable background. Light blue indicates bistable oscillatory state and background state. Striped yellow indicates bistable background and upper fixed point. Red dot marks the interictal standard parameter position used throughout the manuscript. (TIF) [file pcbi.1003787.s005.tif]

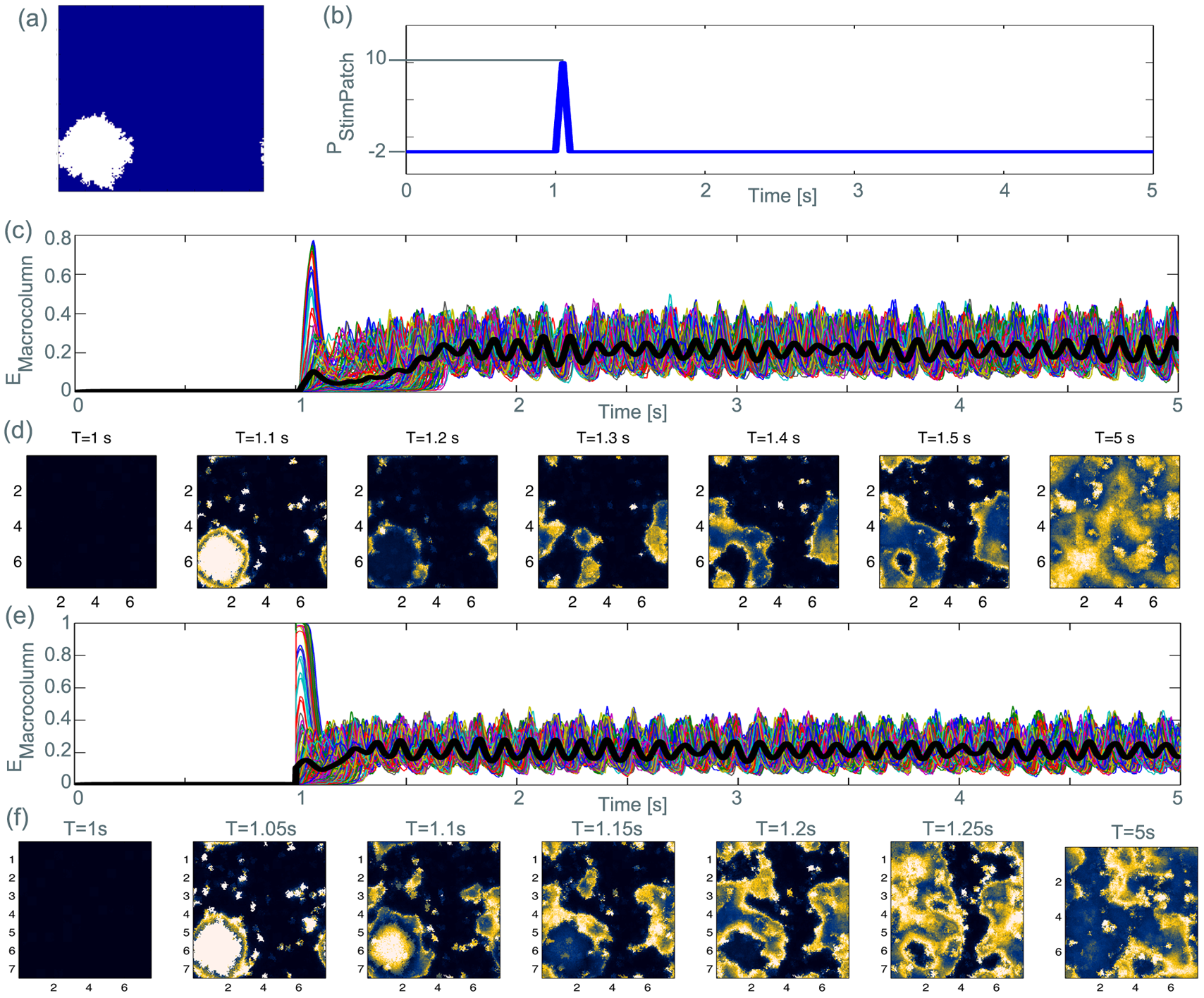

Supplement: Figure S6 — Input pulse vs. initial condition reset pulse. has been used for all simulations, putting the sheet in the bistable regime. (a) Stimulus position on the simulated cortical sheet. This is used for both types of stimuli. (b) The input pulse used to simulate input stimulation (c) and (d). (c) Time series of the average macrocolumn excitatory populations for an input pulse given at T = 1 s. (d) Corresponding snapshots in time for (c). (e) Time series of the average macrocolumn excitatory populations for an initial condition reset to 1 at T = 1 s. (f) Corresponding snapshots in time for (e). (TIF) [file pcbi.1003787.s006.tif]

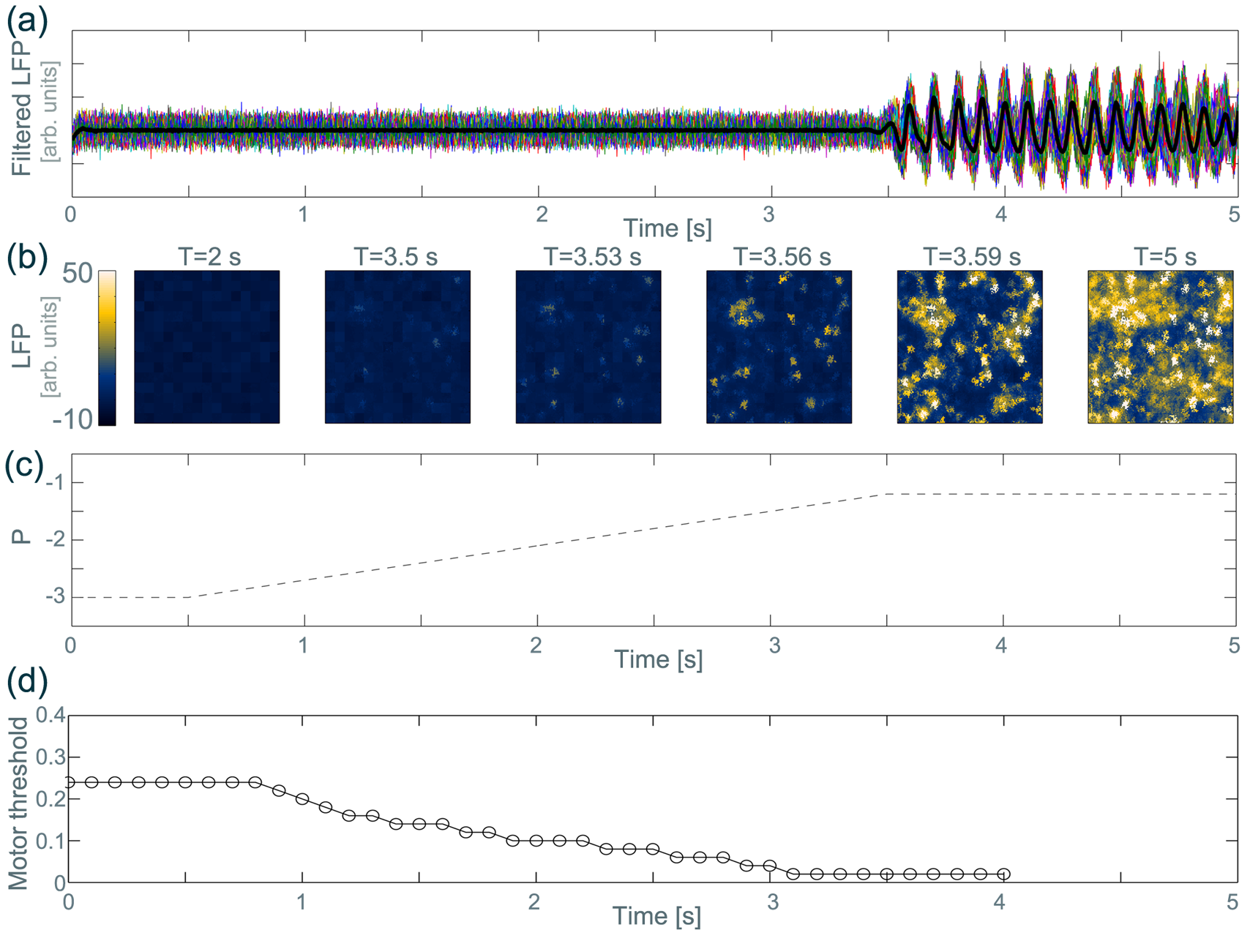

Supplement: Figure S7 — Simulating the changing motor threshold in the lead-up to a seizure. The seizure (see (a,b) for time series) has been induced using a global parameter ramp in (c). The measured motor threshold in the model is shown in (d). (TIF) [file pcbi.1003787.s007.tif]

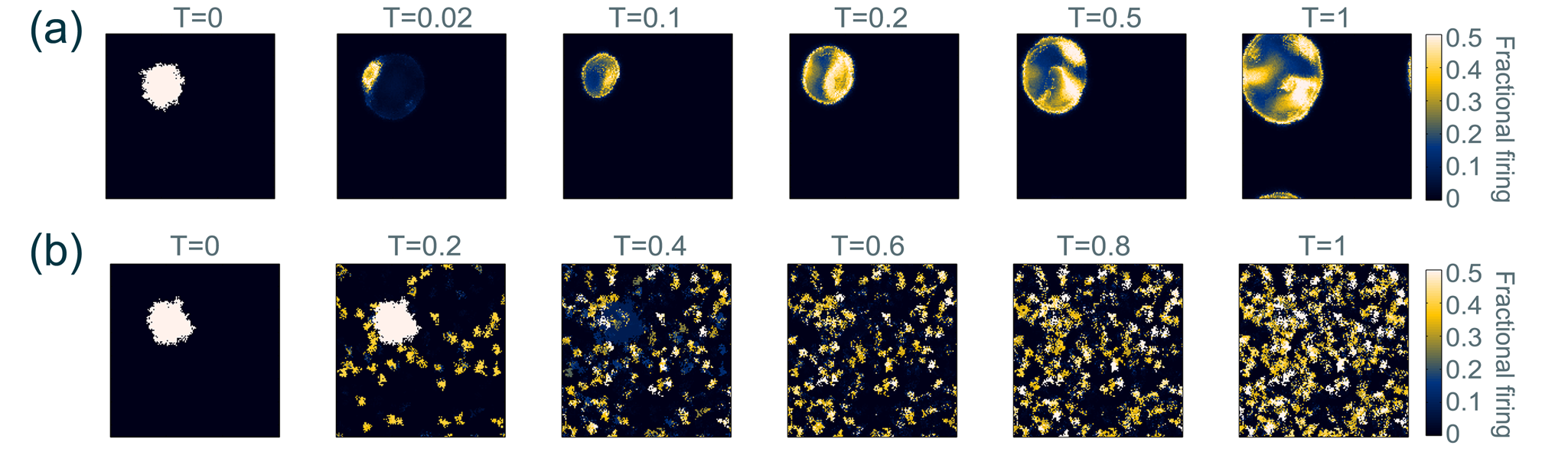

Supplement: Figure S8 — Example propagation patterns using local or remote connections. Snapshot of the sheet at different time points for strong local connection weights, weak remote connection weights (top), and strong remote connection weights and weak local connection weight (bottom). A mixture between both dynamics can be seen in Fig. 5 (d) of the main manuscript. (TIF) [file pcbi.1003787.s008.tif]

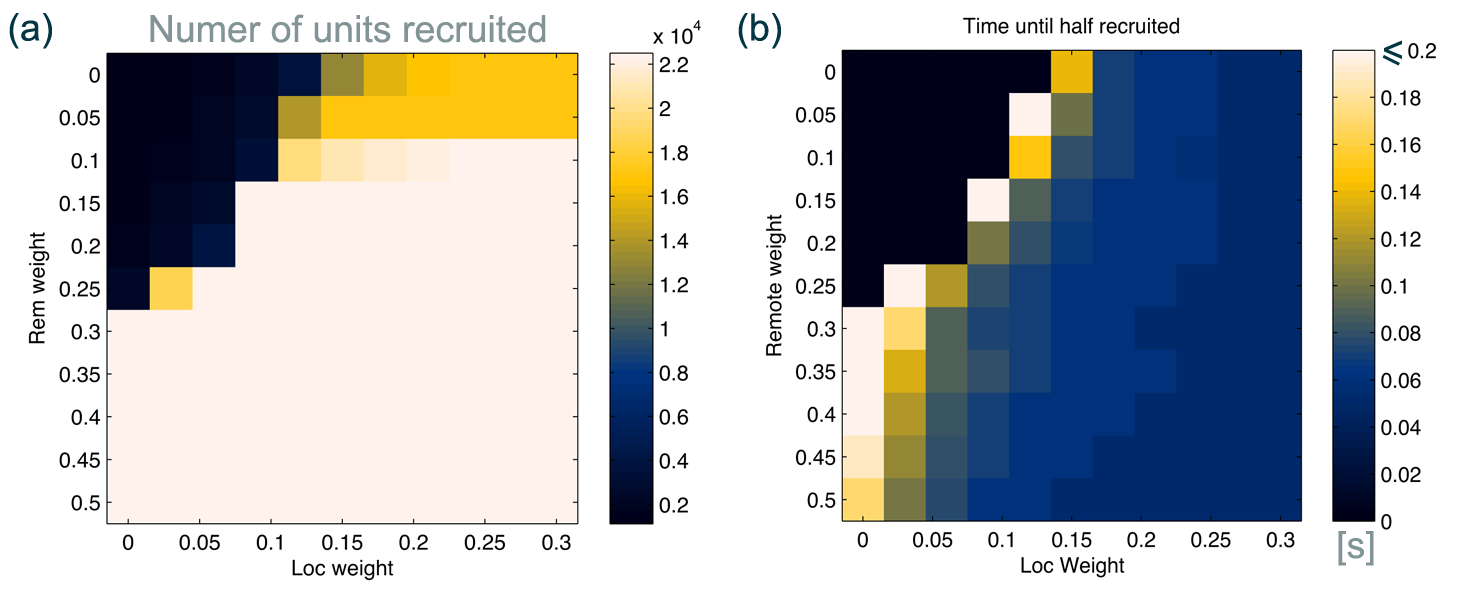

Supplement: Figure S9 — Recruitment dynamics for different local and remote feed-forward connection weights. (a) Number of recruited units 5 s after stimulus, depending on the remote and local connection strength. (b) Time required to recruit half of the final recruitment number as shown in (a). (TIF) [file pcbi.1003787.s009.tif]

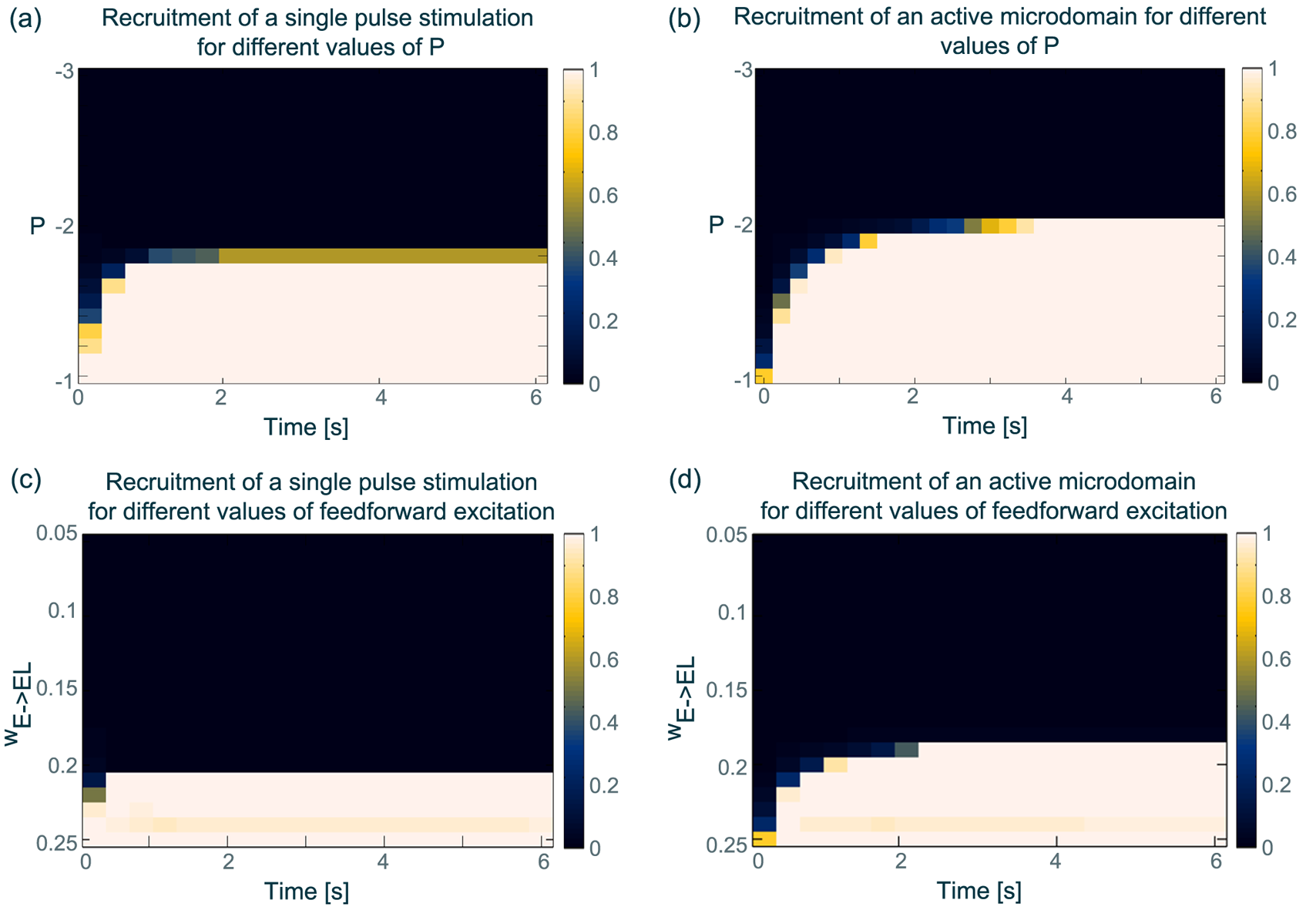

Supplement: Figure S10 — Percentage of recruitment over time and different conditions of the surrounding. (a) For different values of , recruitment (colour code) following a single pulse stimulus was measured every 0.3 s. (b) For different values of , recruitment (colour code) from a hyperactive microdomain was measured every 0.3 s. (c) For different values of , recruitment (colour code) following a single pulse stimulus was measured every 0.3 s. (d) For different values of , recruitment (colour code) from a hyperactive microdomain was measured every 0.3 s. The stimulus location and the location of the hyperactive microdomain were identical in all scans. Each scan point is obtained as the average over 5 different noise inputs. (TIF) [file pcbi.1003787.s010.tif]

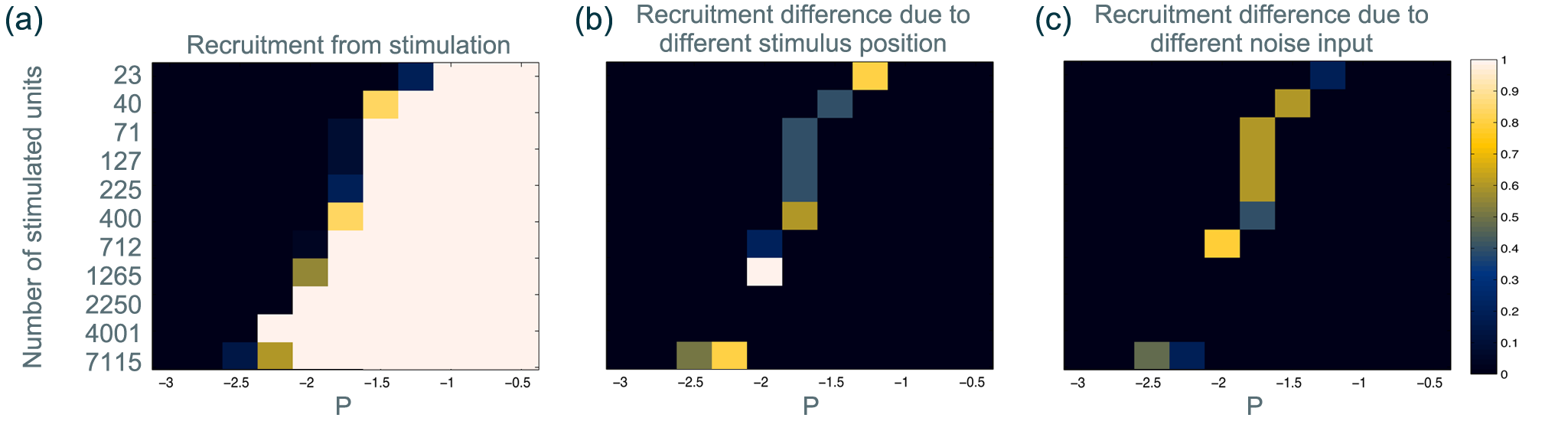

Supplement: Figure S11 — Recruitment depending on and number of units being stimulated. In this scan the stimulus was to contiguous patches on the sheet. (a) Scan result obtained by averaging over different noise inputs, 5 for each of the 5 stimulation positions. The 5 positions were chosen at random. Colour code indicates percentage of recruited units relative to the total number of units. 1 on the colour bar indicated 100% recruitment. (b) The maximum difference in terms of recruitment between different microdomain positions of the same setting (averaged over 5 different noise inputs for each position). (c) The maximum difference in terms of recruitment of the surrounding between the 25 different noise inputs of the same setting, off-setted against the already registered effect of the microdomain position. (TIF) [file pcbi.1003787.s011.tif]

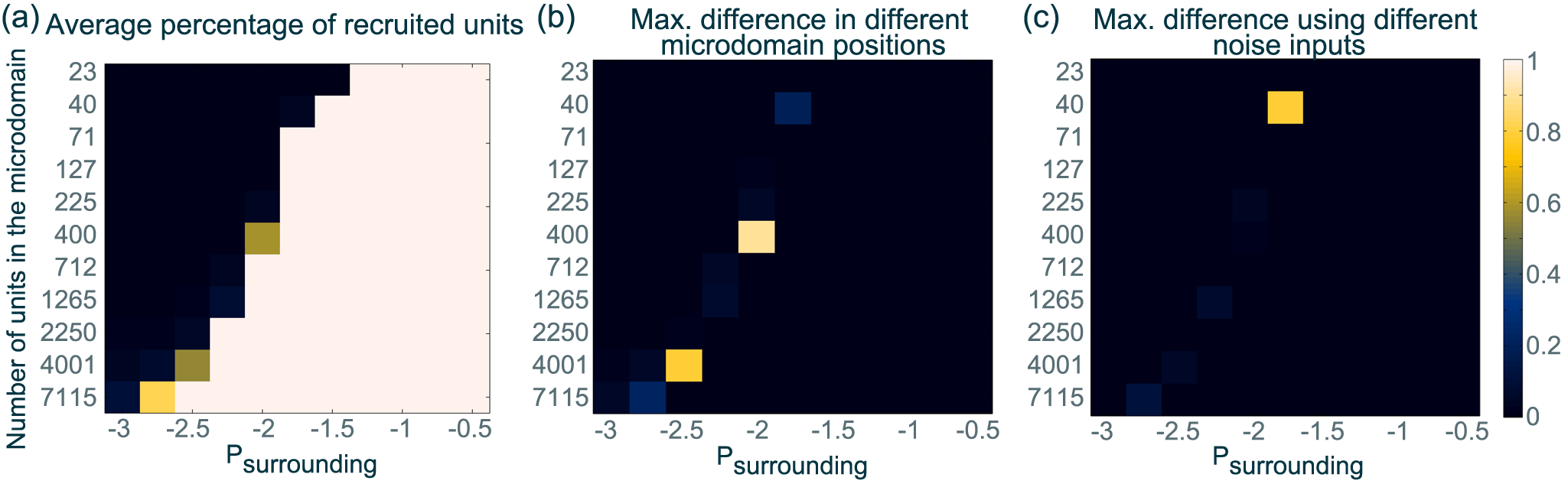

Supplement: Figure S12 — Recruitment depending on and number of units in the hyperactive microdomain. In this scan only one continuous microdomain was used. (a) The scan result is obtained by averaging over different noise inputs, 5 for each of the 5 microdomain positions. The 5 positions were chosen at random. Colour code indicates percentage of recruited units relative to the total number of units outside of the microdomain. 1 on the colour bar indicated 100% recruitment of the surrounding. (b) The maximum difference in terms of recruitment of the surrounding between different microdomain positions of the same setting (averaged over 5 different noise inputs for each position). (c) The maximum difference in terms of recruitment of the surrounding between the 25 different noise inputs of the same setting, off-setted against the already registered effect of the microdomain position. (TIF) [file pcbi.1003787.s012.tif]

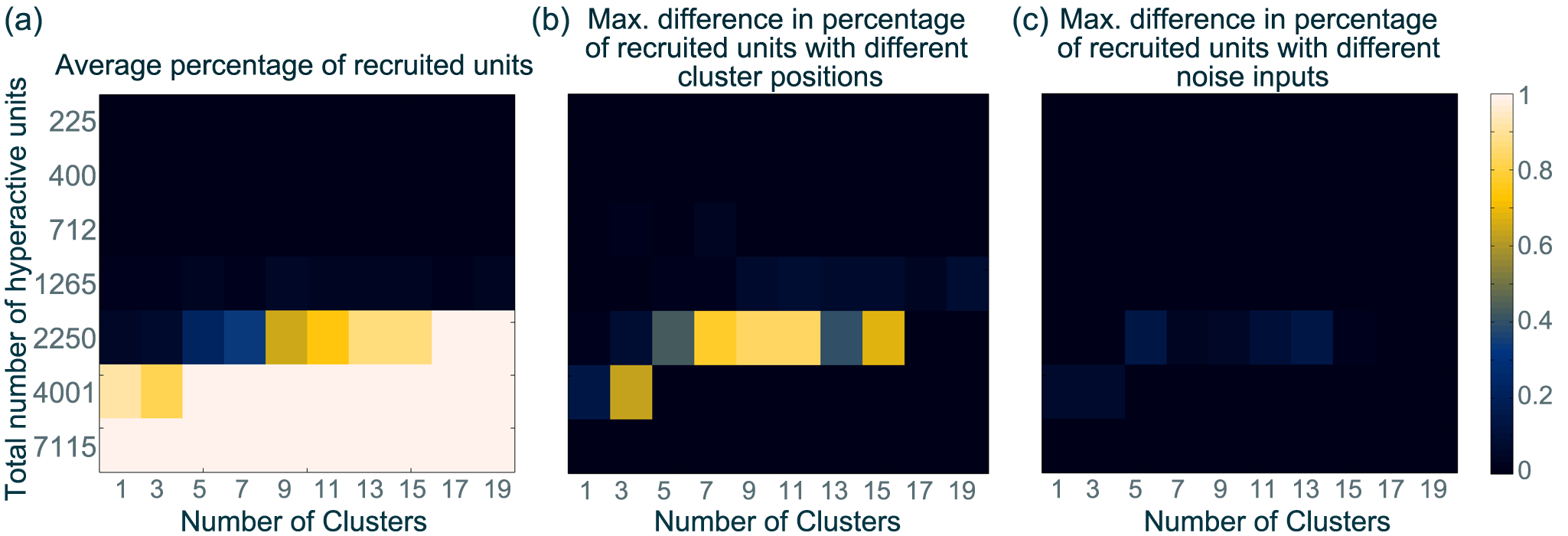

Supplement: Figure S13 — Recruitment depending on the number of subclusters and number of hyperactive units. In this scan was set in the monostable background state. (a) The scan result is obtained by averaging over different noise inputs, 5 for each of the 5 microdomain positions. The 5 positions were chosen at random. Colour code indicates percentage of recruited units relative to the total number of units outside of the microdomain(s). 1 on the colour bar indicated 100% recruitment of the surrounding. (b) The maximum difference in terms of recruitment of the surrounding between different microdomain positions of the same setting (averaged over 5 different noise inputs for each position). (c) The maximum difference in terms of recruitment of the surrounding between the 25 different noise inputs of the same setting, off-setted against the already registered effect of the microdomain position. (TIF) [file pcbi.1003787.s013.tif]

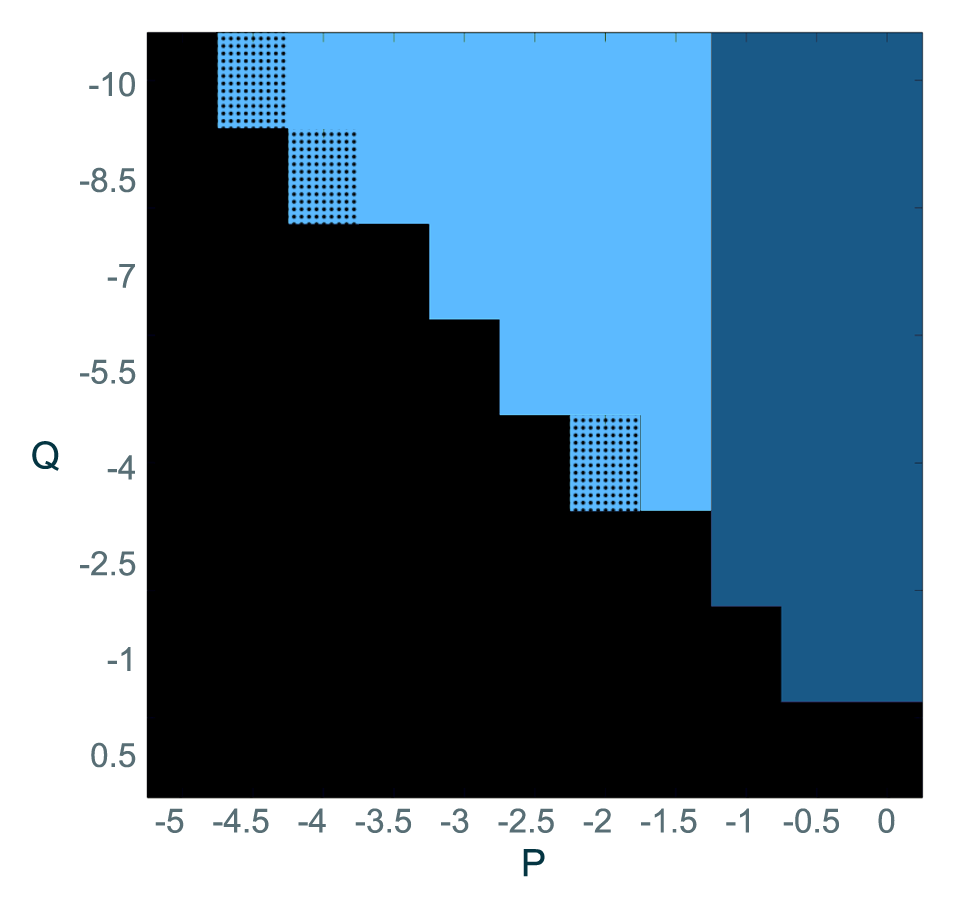

Supplement: Figure S14 — Variation of the bifurcation diagram with changing underlying connectivity. Five different bifurcation diagrams were obtained using five different local and remote connectivities (generated by the same algorithm using the same parameters). Black indicates monostable background. Light blue indicates bistable oscillatory state and background state. Dark blue indicates monostable oscillatory state. Dotted light blue indicates a deviation in at least one of the five scans at this parameter setting. (TIF) [file pcbi.1003787.s014.tif]

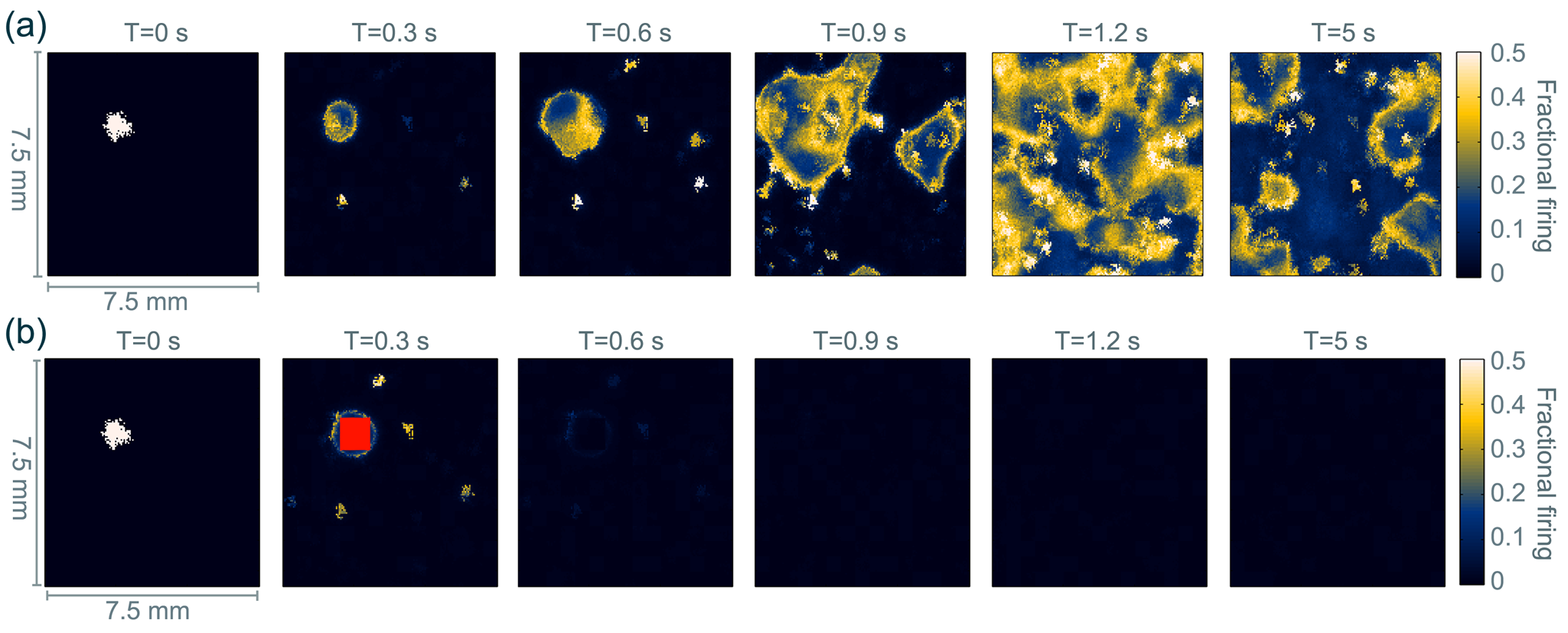

Supplement: Figure S15 — Simulating counter stimulation. (a) Snapshots of fractional firing activity of the populations upon stimulation at . Recruitment starts from a local perturbation. (b) Identical system and simulation conditions as above, only with a counter-stimulus delivered at (red block). The recruitment is suppressed. (TIF) [file pcbi.1003787.s015.tif]

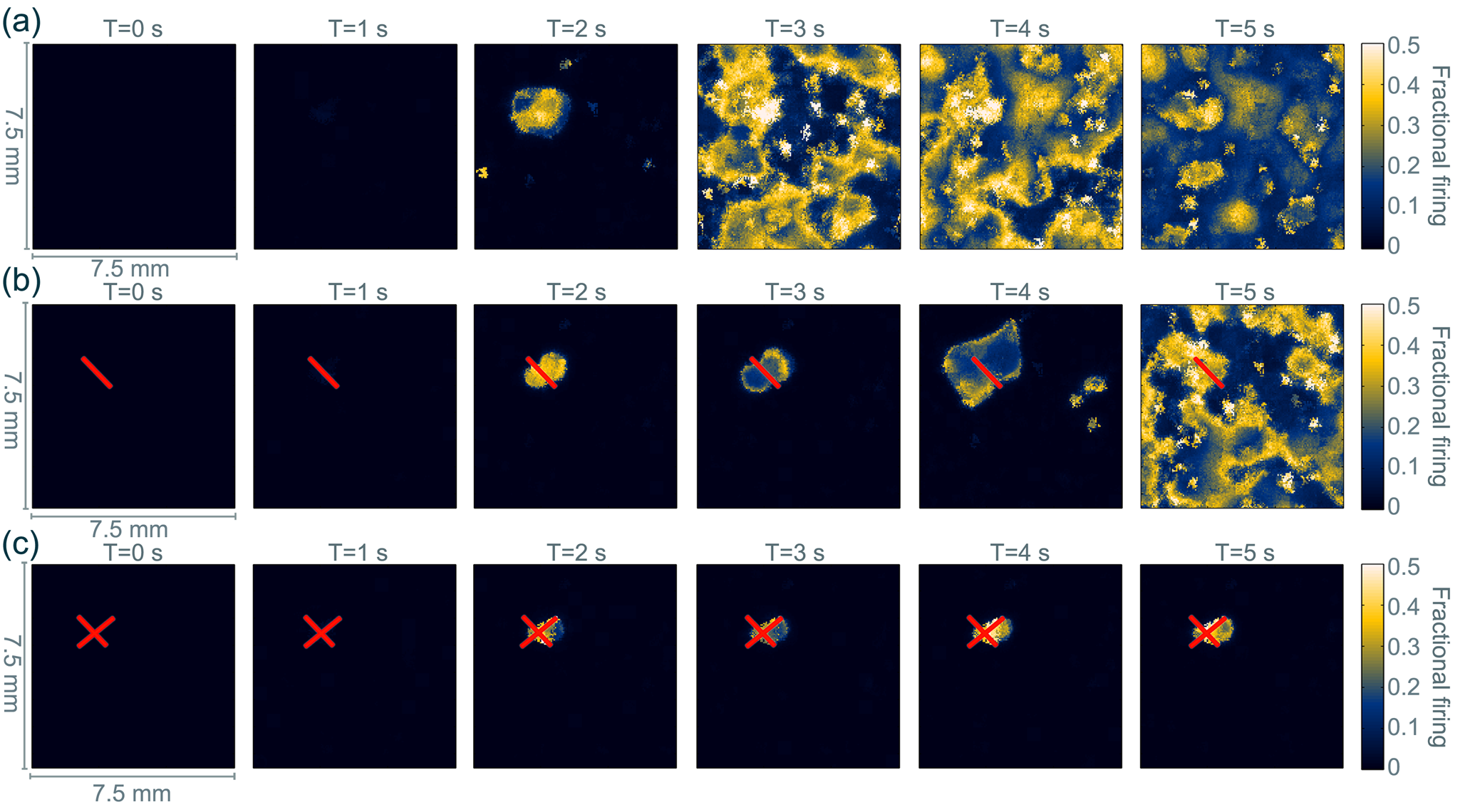

Supplement: Figure S16 — Simulating microincision. (a) Snapshots from a time evolution of fractional firing activity of the populations. Recruitment begins from a hyperactive microdomain (where its value has been ramped to between and ). The surrounding sheet is in the bistable state. (b) Identical system and simulation conditions as (a), only with all connections removed that intersect the cut (red line). Recruitment is delayed and starts from only one half of the microdomain. (c) Identical system and simulation conditions as above, only with an additional cut (second red line). Recruitment is suppressed during the whole simulated time. (TIF) [file pcbi.1003787.s016.tif]

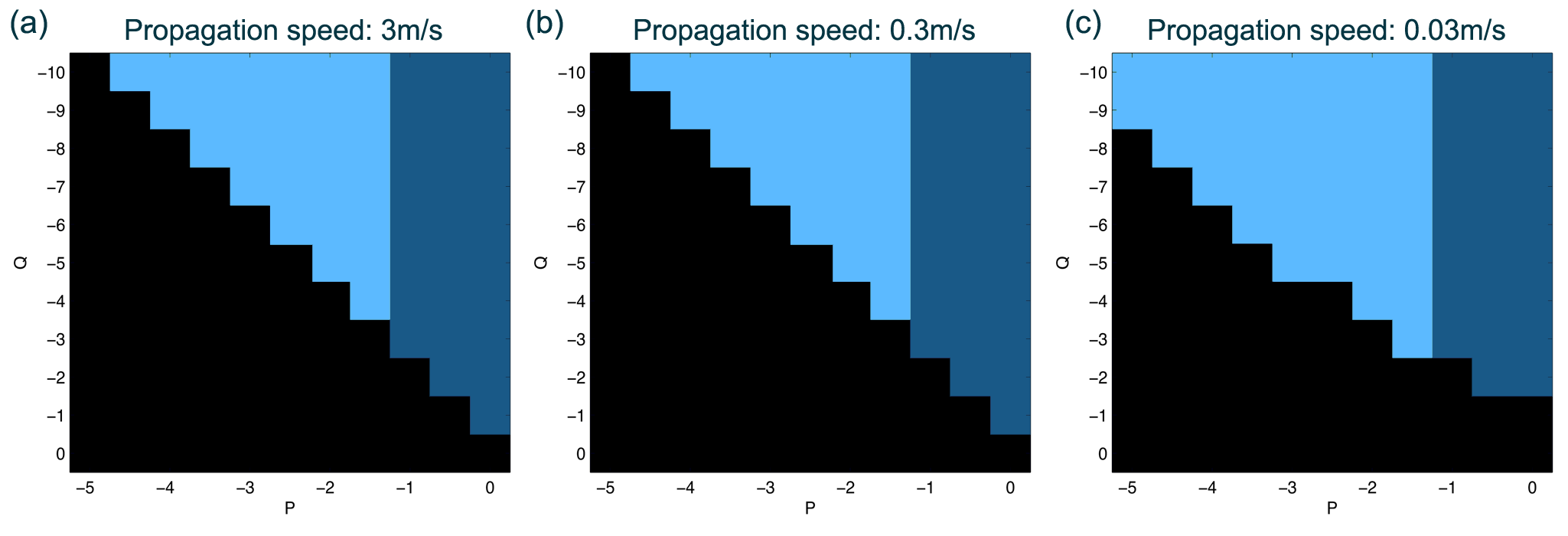

Supplement: Figure S17 — P/Q bifurcation diagram of the whole sheet for different values of the signal propagation speed. Black indicates monostable background. Light blue indicates bistable oscillatory state and background state. Dark blue indicates monostable oscillatory state. (TIF) [file pcbi.1003787.s017.tif]
