# Supplementary material for: Dynamic Mechanisms of Neocortical Focal Seizure Onset
Source: PLoS Comput Biol. 2014 Aug 14;10(8):e1003787. doi: 10.1371/journal.pcbi.1003787 (PMC4133160; doi:10.1371/journal.pcbi.1003787)
Supplement: Text S1 — Additional methods and algorithms. (PDF) [file pcbi.1003787.s018.pdf]

Text S1: Additional methods and algorithms

Local field potential (LFP)

The Wilson-Cowan model does not express the LFP of a minicolumn explicitly as a model variable. In reality the LFP is likely to be some function of the weighted sum of all inputs to the E populations. Hence we approximate the LFP of each unit by the net inputs to the excitatory populations. We ignore volume conduction effects when simulating LFP measured from microelectrodes, and only take the LFP of a local minicolumn. In reality, the LFP measured from a microelectrode will also receive contributions from neighbouring units. As we do not specifically analyse power spectra or waveforms in the current work, this approximation is justifiable. However, future work aiming to model realistic microelectrode LFPs will take into account e.g. the findings of [1].

Multi unit activity MUA

The Wilson-Cowan model explicitly expresses the fractional firing activity of local excitatory and inhibitory populations as variables. In order to show multi unit activity of a minicolumn unit, we calculate the number of spikes of the excitatory population in a time window of $0.1s$. This is done by multiplying the mean percentage firing activity by the estimated number of excitatory neurons (we use 25) in the minicolumn unit. The temporal rasterplot of firing is then plotted time window by time window, using the estimated number of spikes in the window. The parameters (0.1 s and 25 units) used here are only for visualisation of the data in a rasterplot.

Generation of patches

Throughout the manuscript, spatial patches of certain sizes were required, either for stimulation, or for generating heterogeneous domains, or for establishing remote connections to.

All these patches were generated by one algorithm which we describe here. First a single seed unit has to be chosen, either randomly, or specified by x y coordinates on the sheet. We define a cell location matrix M, which is n by n (150 by 150). In M, the seed unit is set to 1, all other units are zero.

The next unit in the patch is obtained by picking a unit according to a probability function. The probability function is a spatial convolution of the cell location matrix M with a 2D Gaussian. This however means that the seed unit itself has the highest likelihood of being picked as the next unit. Hence

the probability function is set to zero at the seed unit location and the whole function is renormalised. The next unit is picked according to this renormalised distribution and also set to 1 in the cell location matrix.

For the subsequent units, the same procedure is followed: Convolution of M with the Gaussian, resetting the probability of the already selected units to zero, and renormalising the remaining probabilities.

This is repeated until the target number of units in the patch is reached.

The width of the Gaussian determines how dispersed the units are in a patch. For the current study we have chosen the width to be very narrow to guarantee dense patches.

Generation of the remote patchy overlapping connectivity

As a first step the sheet is subdivided into macrocolumns (each macrocolumn contains 10 by 10 minicolumns). With n=150, 15 by 15 macrocolumns are defined. These 225 macrocolumns are shuffled into a random order. The first macrocolumn in this order gets 6 remote patches assigned to it (within the defined distance). Each minicolumn in the macrocolumn can choose 104 minicolumns from the 6 patches to connect to. If twice the same source minicolumn chooses the same target minicolumn, the connection is only established once. Hence, the number of outgoing connections of the source minicolumn can be smaller than 104. The subsequent macrocolumn is selected in the list of randomly ordered macrocolumns. This macrocolumn will check if it already has a neighbour that has patches assigned to it. If not it establishes 6 remote patches, as the first macrocolumn. If the macrocolumn already has one or more neighbours, it randomly chooses a neighbouring macrocolumn and picks 3 of its remote patches to connect to, additional to 3 randomly chosen patches within the defined distance. This process is repeated until all minicolumns in all macrocolumns have connected to their patches. In case of zero-flux boundaries, the relative numbers of neighbours within the remote connection distance additionally scales the number of patches of a macrocolumn. I.e. a macrocolumn in the middle of the sheet would have 6 remote patches as usual, but a macrocolumn in the corner of the sheet would only have $0.25 * 6 \approx 2$ remote patches. The remaining 4 patches are registered as off-bound. When sharing connections, these off-bound connections can also be shared. This simulated a cortical sheet that has been cut out of its environment of a bigger continuous cortical sheet.

Properties of the connectivity matrix

Fig. S1 shows the degree distribution, as well as the distance distribution of the connections in the three connectivity matrices $C_{E\rightarrow E_L}$, $C_{E\rightarrow E_R}$, and $C_{E\rightarrow I_L}$. The local connectivity shows a Gaussian (in and out) degree distribution, as expected from a probabilistic distance dependent connectivity scheme. The remote excitatory connection in degree is multi-modal, as each mode represents remote patches that are targeted by a macrocolumn. As there are 100 units in a macrocolumn, and each establishes 104 connections to 6 remote patches, which are 39 units in size, a unit in a remote patch can expect around $\frac{100\times104}{6\times39} = 44.44$ incoming connections (in degree) from one macrocolumn, which is the centre of the first mode (and 88.88 connections from two macrocolumns, i.e. second mode etc.). From the in degree distribution it is clear that remote patches with input from 1, 2, or 3 macrocolumns are fairly frequent. The out degree for remote connections is fixed by the algorithm to 104 and as explained before, the out degree can be slightly smaller than 104. When analysing the distance distribution of all the connections in the local connectivity, as expected the majority of connections is established within $500\mu m$. The rise of numbers of connection towards $200\mu m$ is due to the number of possible neighbours increasing with distance. For the remote connectivity, most connections are within a radius of $3.75mm$ and rapidly decreasing numbers of connections outside this distance.

In reality the out-degree for remote will not be a constant number, but rather a distribution in the population. This could be incorporated and investigated in future studies.

References

1. Lindén H, Tetzlaff T, Potjans TC, Pettersen KH, Grn S, et al. (2011) Modeling the spatial reach of the LFP. Neuron 72: 859–872.
