# Supplementary material for: Dynamic Mechanisms of Neocortical Focal Seizure Onset
Source: PLoS Comput Biol. 2014 Aug 14;10(8):e1003787. doi: 10.1371/journal.pcbi.1003787 (PMC4133160; doi:10.1371/journal.pcbi.1003787)
Supplement: Text S2 — Effect of boundary conditions. (PDF) [file pcbi.1003787.s019.pdf]

Text S2: Boundary effects

The choice of toroidal boundaries and zero-flux boundaries both come with their advantages and drawback, and both are approximations to the conditions on the cortex. We used toroidal boundaries throughout the main manuscript. Here we shall compare some results using zero-flux boundaries to our results presented in the Results section.

The class I and class II global conditions (oscillatory and bistable) are still found in very similar parameter regions of the model and the propagation speed and pattern stay preserved qualitatively, with some minimal quantitative variations especially near the boundary (data not shown). This is intuitively understandable as seizure onset is supported by the global conditions. The class III onset type however is driven by local processes and local connectivity, and hence we investigate the zero-flux boundary condition on class III in more detail.

Class III w.r.t. boundary conditions

In order to investigate the effect of the boundary conditions on the class III seizures, we have rescanned the average percentage of recruitment with respect to the total number of hyperactive units and the number of subclusters using zero-flux boundaries (Fig. S2 (a)). This is the same scan as in Fig.8 (a) in the main MS, only using zero-flux boundaries. It is clear that the level of recruitment is in general lower and significant recruitment is only noticeable at a higher number of total hyperactive units. This is to be expected as the recruitment only invades the area between the hyperactive clusters. With zero-flux boundaries we assume that the simulated sheet is cut off any other neighbouring input. Hence recruitment is restricted to the total region spanned by the subclusters. No recruitment occurs towards the edge of the simulated sheet (see. Fig. S2 (d)). With toroidal boundaries, 100% recruitment could happen due to the recruitment across the torus “boundaries”. Hence the scans using toroidal boundaries are overestimations of recruitment, if the hyperactive units are indeed restricted to the ones being simulated. However, if assuming a similar subcluster density in the neighbourhood of the simulated sheet as on the sheet, the estimations using toroidal boundaries are valid. Depending on the exact research question either of the two boundary conditions, or simply a bigger system can be used. For our purpose (demonstrating the actual mechanism of recruitment from a network of hyperactive units on monostable background), either boundary condition can be used and does not affect the suggested mechanism.
