# Supplementary material for: Dynamic Mechanisms of Neocortical Focal Seizure Onset
Source: PLoS Comput Biol. 2014 Aug 14;10(8):e1003787. doi: 10.1371/journal.pcbi.1003787 (PMC4133160; doi:10.1371/journal.pcbi.1003787)
Supplement: Text S3 — Parameter setting for the single unit. (PDF) [file pcbi.1003787.s020.pdf]

Text S3: Parameter setting for the single unit

The parameters of the single unit were chosen such that the single unit on its own (without any external input) would only display background behaviour (lower fixed point). However, the parameters were set near an oscillatory parameter region. This enabled the single unit, once receiving input from neighbours, to be brought into oscillatory behaviour. Fig. S3 shows the bifurcation behaviour of the single unit with and without noise input. The red dot represents its current parameter setting. Given a constant excitatory input from neighbours at the current parameter setting, which equates an increase in P, the single unit would change from a node to a focus (Fig. S4). Hence, in the noise driven system oscillations of increasing amplitude can be observed (Fig. S3 (b)).

However, the input from neighbours is more complex than a constant excitatory input and hence the single unit dynamics are only of limited use for understanding the spatially extended system. Hence we conducted extensive parameter scans to identify the parameter regions for fixed point and oscillatory (or bistable) behaviour in the first Results section. The point we underline here though is that the single unit in its standard setting (red dot) is unable to generate oscillations.
