# Supplementary material for: Dynamic Mechanisms of Neocortical Focal Seizure Onset
Source: PLoS Comput Biol. 2014 Aug 14;10(8):e1003787. doi: 10.1371/journal.pcbi.1003787 (PMC4133160; doi:10.1371/journal.pcbi.1003787)
Supplement: Text S4 — Additional parameter scans for the full system. (PDF) [file pcbi.1003787.s021.pdf]

Text S4: Parameter scans for the full system

We complement the parameter scans of the first Result section with additional scans of other parameters (Fig. S5). These are not shown in the main body of the manuscript, as the added information is limited, but are shown here for completeness. Current parameter positions are marked with red dots. All scans are conducted assuming homogeneous parameter settings for all units. The only heterogeneity in the system is the connectivity, which is described in Methods and in Text S1.

Fig. S5 shows that the current parameter setting in the self excitation and inhibition parameters are also near the bistability of background (lower fixed point) and oscillations (light blue).
