# Supplementary material for: Dynamic Mechanisms of Neocortical Focal Seizure Onset
Source: PLoS Comput Biol. 2014 Aug 14;10(8):e1003787. doi: 10.1371/journal.pcbi.1003787 (PMC4133160; doi:10.1371/journal.pcbi.1003787)
Supplement: Text S5 — Details of parameters and simulation methods for every figure. (PDF) [file pcbi.1003787.s022.pdf]

Text S5: Details of parameters and simulation methods for every figure

The model parameters used to produce the figures in the current work all follow one basic set of parameters. This basic set of parameters puts the system in a monostable background state globally, although near to a globally bistable state. Details of the meaning of the parameters can be found in the Methods section. Here we shall list the basic set of parameters (see Tab. 1) and describe in detail how each figure has been obtained.

Table 1. Basic set of parameters used throughout the whole manuscript, if not stated otherwise.

Parameter name	Meaning	value
P	Input to excitatory population	-2.5
Q	Input to inhibitory population	-5
C_S	Standard deviation of noise input	1
a	determines steepness of the Sigmoid activation function	1
θ	Offset of the Sigmoid activation function in terms of input	4
$w_{E \to E_{self}}$	Self excitation of excitatory population	10
$w_{E \to E_L}$	Local neighbour excitation from excitatory population	0.15
$w_{E \to E_R}$	Remote neighbour excitation from excitatory population	0.05
$w_{E \to I_{self}}$	Excitation of inhibitory population in the same unit	15
$w_{E \to I_L}$	Local neighbour excitation of inhibitory population from excitatory population	0.1
$w_{I \to E}$	Inhibition of excitatory population in the same unit	25
$w_{I \to I}$	Inhibition of inhibitory population in the same unit	0
τ_E	Time constant of excitatory population	0.04
τ_I	Time constant of inhibitory population	0.02

Fig. 3: In order to determine the global behaviour of the system at different parameter settings of P and Q, we systematically vary P and Q in each unit. We use zero initial conditions and run the system for 1.5 s. We calculate the mean time series (averaging over all units) and determine the mean (m_0) and standard deviation (s_0) of this mean time series in the last 0.75 s. In the next step we initialise the sheet with the state of the sheet at the last time step of the previous simulation. Subsequently we use initial condition reset stimuli of different sizes and random locations to perturb the sheet and simulate for another 1.5 s. The different stimulation sizes were 0.005, 0.02, 0.04, 0.06, 0.08, 0.1, 0.12, 0.14, 0.3, and 1 (expressed as fraction of units relative to the total number of units). After each stimulus, the mean time series was calculated, the mean ($m_{0.005}, m_{0.02}, ...$) and standard deviation ($s_{0.005}, s_{0.02}, ...$) of the last 0.75 s of the mean time series was obtained. As a final step, the maximal mean and standard deviation was chosen from $m_{max} = max(m_{0.005}, m_{0.02}, ...)$ except for m_0 (also $s_{max} = max(s_{0.005}, s_{0.02}, ...)$ except for s_0). The procedure was repeated for all scanned values of P and Q. Every simulation used a different noise vector as subcortical input. To plot the bifurcation diagram, we determined the monostable background state

(black) by $m_{max} - m_0 \leq 0.05$ and $s_0 \leq 0.001$ and $s_{max} \leq 0.001$. The bistable background and oscillatory state (light blue) fulfils $m_{max} - m_0 > 0.05$ and $s_0 \leq 0.001$ and $s_{max} > 0.001$. The monostable oscillatory state (dark blue) fulfils $m_{max} - m_0 \leq 0.05$ and $s_0 > 0.001$ and $s_{max} > 0.001$. The bistable background and upper fixed point (striped yellow) fulfils $m_{max} - m_0 > 0.05$ and $s_0 \leq 0.001$ and $s_{max} \leq 0.001$.

Fig. 4: A patch of the size 225 units (1%) has been selected in the centre of the simulated sheet to receive 1.5 times stronger feed forward excitation than the rest of the sheet, to create an obvious heterogeneity in the sheet. As such, the connectivity of the sheet is already heterogeneous due to the nature of the local and remote patchy connectivity. The additionally created heterogeneity is only there to clearly demonstrate the point of earlier reaction to a global parameter change. The global parameter change in this case ramps $P = -3$ to $P = -1$ between T=0.5 s to 4 s. Total simulation time was 5 s. The LFP was obtained as described in the subsection "Local field potential" in Text S1.

Fig. 5: In order to achieve an overall bistable condition on the sheet, the parameter $w_{E \to I_L}$ has been changed to 0.05 (from 0.1). The trigger stimulus was 5% of the total units. In (c) the raster plot of firing rate was generated as stated in the following subsection "Multi unit activity".

Fig. 6: In (d) and (e) the surrounding system is not in the bistable setting ($P_{surrounding} = -2.5$). The microdomain has been ramped into the monostable oscillatory region ($P_{microdomain} = -2.5$ at 0-0.5 s, ramped to $P_{microdomain} = 1$ at 0.5-1.5 s, remains at $P_{microdomain} = 1$ from 1.5 s until 5 s). In (f) and (g) the surrounding system is in the bistable setting ($P_{surrounding} = -1.9$). The microdomain behaves the same as in (d) and (e).

Fig. 7 (a) and (b) : Two different $P_{surrounding}$ were used: (a) $P_{surrounding} = -2.5$, (b) $P_{surrounding} = -2.8$. The hyperactive clusters were generated by setting $P_{microdomain} = 0$. The number of recruited units is determined by counting the number of units outside the oscillatory microdomains that have become oscillatory after 3 s simulation time. The percentage of recruited units is calculated base on the number of units not in the microdomain that have become oscillatory. The average percentage was calculated based on 5 different randomly generated microdomains of the same size (number of units), and each microdomain was simulated with 5 different noise inputs. I.e. 25 different noise inputs and 5 different microdomains were used for the average recruitment percentage at each scanned parameter point.

Fig. 7 (d) and (f) : $P_{surrounding} = -2.5$ was used for both simulations. The hyperactive clusters were generated by ramping $P_{microdomain}$ from -2.5 to 0 between T=0.5 s and T=1.5 s. From T=1.5 s onwards, $P_{microdomain} = 0$.

Fig. 8 (a): $P_{surrounding} = -2.5$. We use zero initial conditions and run the system for 2 s. We calculate the percentage of recruited units in the microdomain R_0 (relative to the size of the microdomain). In the next step we initialise the sheet with the state of the sheet at the last time step of the previous simulation. Subsequently we use a single-pulse stimulus of the same size and location as the microdomain to stimulate the microdomain. This was simulated for another 2 s, the percentage of recruited units in the microdomain after the stimulus (R_{stim}) was obtained. This process was repeated with 5 different noise inputs each for 5 different microdomain positions and the maximal values from the 25 repeats of R_{stim}, R_0 was stored as Rm_{stim} and Rm_0. If $Rm_0 < 1$ and $Rm_{stim} = 1$ then we deem the microdomain bistable. If $Rm_0 < 1$ and $Rm_{stim} < 1$ then the microdomain is monostable in the background state. If $Rm_0 = 1$ and $Rm_{stim} = 1$ then the microdomain is monostable in the oscillatory state. We also checked that none of the recruited units reached the permanently firing state. The procedure was repeated for all scanned values of $P_{microdomain}$ and the size of the microdomain.

Fig. 8 (c) : $P_{surrounding} = -2.5, P_{microdomains} = -0.5$. Repeated stimuli of size 1% were delivered at T=0,3,6,9,12,15,18,21 s. Stimuli were generated randomly. The subclusters of microdomains were generated using 40 subclusters, each with 50 hyperactive units (total number of hyperactive units: 2000, or 8.9% of the whole system).

Supplementary figures

Fig. S1: Scan and time series obtained in the same way as Fig. 7 in the main MS, only using zero-flux boundary conditions on the system.

Fig. S2: A single unit was simulated using the same standard parameter set. Parameter scan was performed forward and backward in P for each $w_{E \to E_S}$. Oscillations were detected by taking the difference in maximum and mininum of 5 s of the time series after an initial 5 s simulation time.

Fig. S3: Scan performed in the same way as Fig. 3, only using different parameters.

Fig. S4: $P_{Surrounding} = -2$. (e,f) used a initial condition reset as described before. (c,d) used a parameter change in the stimulated units P_{Stim}. The parameter change is shown in (b).

Fig. S5: Described in the corresponding text in detail.

Fig. S6: $w_{E \to I_L} = 0.05, P = -2.5$ homogeneously, otherwise the standard parameter set was used. Stimulus was delivered using an initial condition reset to 1 in the E variable of the stimulated units. (a) $w_{E \to E_L} = 0.22, w_{E \to E_R} = 0.01$ (b) $w_{E \to E_L} = 0.005, w_{E \to E_R} = 0.165$

Fig. S7: $w_{E \to I_L} = 0.05, P = -2.5$ homogeneously, otherwise the standard parameter set was used. Number of units recruited was determined after 5 s of simulation time. The same stimulus (5% of the sheet) was used for all scan points.

Fig. S8: Parameters being scanned are indicated on the y-axis. Otherwise the standard parameter set was used. We used 225 units in a contiguous cluster in the middle of the sheet for both the temporary stimulus as well as for the oscillatory microdomain. The figure shows the average over 5 noise inputs in each scanned parameter point.

Fig. S9: Single continuous microdomains have been used for this scan. Parameter $P_{surrounding}$ varied (x-axis) and different sizes of stimuli have been scanned. The number of recruited units is determined by counting the number of units that have become oscillatory after 3 s simulation time. The percentage of recruited units is calculated based on the number of units not in the microdomain that have become oscillatory. The average percentage was calculated based on 5 different randomly generated microdomains of the same size (same number of units) and each microdomain was simulated with 5 different noise inputs each. I.e. 25 different noise inputs and 5 different microdomains were used for the average recruitment percentage at each scanned parameter point. The variance introduced by the stimulus position (2nd panel) was determined by the maximum difference in recruitment following the 5 tested positions. The variance introduced by the noise (3rd panel) was determined by the maximum difference in recruitment following the 25 tested noise input, off-setted by the already determined variance introduced by the stimulus position.

Fig. S10 : Single continuous microdomains have been used for this scan. Parameter $P_{surrounding}$ varied (x-axis) and different sizes of microdomains have been scanned. The microdomain was generated by setting $P_{microdomain} = 0$. The number of recruited units is determined by counting the number of units that have become oscillatory after 3 s simulation time. The percentage of recruited units is calculated based on the number of units not in the microdomain that have become oscillatory. The average percentage was calculated based on 5 different randomly generated microdomains of the same size (same number of units) and each microdomain was simulated with 5 different noise inputs each. I.e. 25 different noise inputs and 5 different microdomains were used for the average recruitment percentage at each scanned parameter point. The variance introduced by the microdomain position (2nd panel) was determined by the maximum difference in recruitment following the 5 tested positions. The variance introduced by the noise (3rd panel) was determined by the maximum difference in recruitment following the 25 tested noise input, off-setted by the already determined variance introduced by the microdomain position.

Fig. S11: $P_{surrounding} = -2.5$. The hyperactive clusters were generated by setting $P_{microdomain} = 0$. The number of recruited units is determined by counting the number of units that have become oscillatory after 3 s simulation time. The percentage of recruited units is calculated based on the number of units not in the microdomain that have become oscillatory. The average percentage was calculated

based on 5 different randomly generated microdomains of the same size (number of units) and each microdomain was simulated with 5 different noise inputs each. I.e. 25 different noise inputs and 5 different microdomains were used for the average recruitment percentage at each scanned parameter point. The variance introduced by the microdomain position (2nd panel) was determined by the maximum difference in recruitment following the 5 tested positions. The variance introduced by the noise (3rd panel) was determined by the maximum difference in recruitment following the 25 tested noise input, off-setted by the already determined variance introduced by the microdomain position.

Fig. S12: Scans followed the same protocol as Fig. 3 (a) in the main manuscript. 5 different connectivities generated by the same algorithm and connection parameters were used. Dotted parameters indicate where a difference in at least one of the 5 connectivities existed in the scan results.

Fig. S13: $P_{surrounding} = -1.7$. Stimulus size was 1% of the whole system. Counter stimulus was delivered by resetting the initial conditions to zero in E and I.

Fig. S14 : $P_{surrounding} = -1.9$. Microdomain size was 1% of the whole system. $P_{microdomain}$ was ramped from -1.9 to 1 from T=0.5 s to T=1.5 s.

Fig. S15: Scans followed the same protocol as Fig. 3 (a) in the main manuscript. The system including delays was simulated using the Euler-Maruyama method, only taking into account the delay time for each connection.
