# Supplementary material for: Dynamic Mechanisms of Neocortical Focal Seizure Onset
Source: PLoS Comput Biol. 2014 Aug 14;10(8):e1003787. doi: 10.1371/journal.pcbi.1003787 (PMC4133160; doi:10.1371/journal.pcbi.1003787)
Supplement: Text S6 — Simulating stimulation. (PDF) [file pcbi.1003787.s023.pdf]

Text S6: Simulating stimulation

Pulse stimuli were simulated in the manuscript by resetting the value of the E population in the target units to 1, essentially performing a initial condition reset at the stimulation time. From the dynamical systems perspective, this ensures that only the phase space of the system is explored for potential bistabilities, which is what these pulse stimuli were mainly used for in the manuscript.

However, when for instance comparing results such as the propagation pattern upon pulse stimuli to actual data such as [1], one might argue that simulating the stimulation pulse as an input pulse (given to P) is required. However in our system, Fig. S6 shows that both types of stimulation (initial condition reset and input pulse) give the same overall qualitative behaviour of a propagating wavefront of recruitment. The recruitment route is also highly comparable (e.g. compare 6th panel of Fig. S6 (d) with 5th panel of Fig. S6 (e)). The main noticeable difference between the two types of stimuli is that the initial rise of activity is captured in the input stimulus, whereas the initial condition reset stimulus essentially assumes instant rise in activity. However, the details of rise of activity do not affect the results presents in this work and is perhaps more interesting for a modelling study concerned with the details of the nature of stimulation. As the details of the stimulus are not the scope of the current work, we only use initial condition resets in the main manuscript.

Using the initial condition reset, we can also simulate a global perturbation, by stimulating all units at once. Such a global perturbation could be for instance a TMS pulse. To simulate the motor threshold as measured in clinical scenarios by TMS [2], we increase the E and I value of all units on the sheet by the stimulus amplitude. This is justified as the TMS pulse has wide-spread effects spatially and we assume an effect on both excitatory and inhibitory populations . We gradually increase the stimulus amplitude (from 0.02 in steps of 0.02 to 0.4) and measure the spatially averaged time series of the whole sheet. If after certain stimulus amplitude the spatially averaged time series crosses a arbitrarily chosen threshold (a measure for whether a hypothetical motor response was elicited or not, here we use 0.225), we deem this stimulus amplitude to be the motor threshold.

When applying this measure of motor threshold to a simulation, where P is globally ramped into a monostable oscillatory state, we find that the motorthreshold can decrease with the ramping of P (see Fig. S7). The stimulus strength required to induce the mean activity of the whole sheet to cross a threshold was measured and compared to the clinical findings of the decreased TMS motor-threshold [2]. Fig. S7 (d) shows that the simulated motor-threshold decreases before the seizure onset. The decrease in the simulated motor threshold is due to the increase in P that increases the basal level of activity on the sheet, thus leading to a higher TMS response. Thus we confirm that the model correctly reflects the results in [2].

References

1. Schevon CA, Weiss SA, McKhann G, Goodman RR, Yuste R, et al. (2012) Evidence of an inhibitory restraint of seizure activity in humans. Nat Commun 3: 1060.

2. Badawy R, Macdonell R, Jackson G, Berkovic S (2009) The peri-ictal state: cortical excitability changes within 24 h of a seizure. Brain 132: 1013-1021.
