# Supplementary material for: Dynamic Mechanisms of Neocortical Focal Seizure Onset
Source: PLoS Comput Biol. 2014 Aug 14;10(8):e1003787. doi: 10.1371/journal.pcbi.1003787 (PMC4133160; doi:10.1371/journal.pcbi.1003787)
Supplement: Text S7 — Propagation patterns and recruitment speed. (PDF) [file pcbi.1003787.s024.pdf]

Text S7: Propagation pattern, propagation speed

A: Propagation pattern on the bistable sheet

When using a low connection weight for remote connections and a high connection weight for local connections (Fig. S8 (a)), the wavefront becomes more even and approximates the spatially continuous case as shown in [1]. Conversely, when using a high connections weight for remote connections and a low weight for local connections, a patchy activation pattern can be observed, which only activates scattered patches, but covers a significantly bigger distance in the same time. Such a pattern is in good agreement with the clinical observation of only a subset of neurons participating in the seizure event by Truccolo *et al.* (2011) [2]. Both propagation types can and probably do happen. It might be that very near to the "focus" some local parameters are changed, like Schevon *et al.* (2012) suggests and recruitment propagates as a wave. However, much further away regions are probably recruited via remote or long-distance connections (first) and activation is primarily patchy. Hence, the conflicting recruitment dynamics described by Truccolo *et al.* (2011) and Schevon *et al.* (2012) is explained in our model by the propagation of activity via different networks.

When scanning remote and local connection weight systematically, we confirm the observation shown in the simulations in Fig. S8. Although recruitment through local connections eventually excites all units, the time it takes to do so is slower than to recruit via remote connections (or a combination of both), see Fig. S9.

B: Recruitment speed

In order to investigate the class II recruitment speed with respect to the parameter setting of the surrounding, we computed the percentage of recruitment in 0.3 s intervals. The exemplary parameters we chose to scan were $P_{surrounding}$ and $w_{E \to E_L}$. We also compared the nature of the stimulus (single pulse stimulus or a hyperactive microdomain). For this scan we kept the stimulus location (a small contiguous patch consisting of 225 units in the middle of the sheet) the same for all scan conditions. We used 5 different noise inputs for each scan condition and averaged over them for the resulting percentage of recruitment. The noise itself hardly affected the scan result (data not shown). The scan results can be seen in Fig. S10.

The general property of microdomains to recruit at a slightly lower level of excitation (compare Fig. S10 (a) with (b), and (c) with (d)) of the surrounding as pointed out in the main text is reconfirmed here. Interestingly, this additional recruitment at slightly lower levels of excitation is often progressing slowly (up to 4 s until full recruitment). The source of this effect is unknown, but most likely related to the effectiveness of repeated (oscillatory) coherent stimulus provided by a microdomain, as opposed to a brief synchronous pulse provided by a single pulse stimulus.

This section demonstrates that a range (from 4 s to under 0.3 s for full recruitment) of possible recruitment speeds can be observed depending on the nature of the pulse and the setting of surrounding. In fact the fastest full recruitment in the system can happen within 0.04 s (see Fig. S9). We can estimate the propagation speed by assuming the average distance to either side of the sheet from the patch in the middle of the sheet to be about 4 mm. Hence the recruitment speed can range between 1 mm/s to 100 mm/s using the standard parameter setting and the two parameters (P and $w_{E \to E_L}$) that we chose to vary. This means the propagation speed can span at least two orders of magnitude in our model, which is in agreement with experimental evidence [3].

References

1. Kim JW, Roberts JA, Robinson PA (2009) Dynamics of epileptic seizures: evolution, spreading, and suppression. J Theor Biol 257: 527–532.

2. Truccolo W, Donoghue JA, Hochberg LR, Eskandar EN, Madsen JR, et al. (2011) Single-neuron dynamics in human focal epilepsy. Nat Neurosci 14: 635-641.

3. Trevelyan AJ, Sussillo D, Yuste R (2007) Feedforward inhibition contributes to the control of epileptiform propagation speed. J Neurosci 27: 3383-3387.
