# Supplementary material for: Dynamic Mechanisms of Neocortical Focal Seizure Onset
Source: PLoS Comput Biol. 2014 Aug 14;10(8):e1003787. doi: 10.1371/journal.pcbi.1003787 (PMC4133160; doi:10.1371/journal.pcbi.1003787)
Supplement: Text S8 — Additional parameter scans and their variability. (PDF) [file pcbi.1003787.s025.pdf]

Text S8: Additional scan results and their variance

A: Single pulse stimuli on a bistable sheet

Fig. S11 investigates the relationship between P and number of units targeted a single pulse stimulus. From this scan it becomes clear that the stimulus size required to elicit a full recruitment has to grow with decreasing P (Fig. S11 (a)). When the sheet becomes monostable at $P = -2.5$, no stimulus can trigger full recruitment (although some partial recruitment can be registered). We also show the variance of this scan result depending on the position of the stimulus (Fig. S11 (b)) and the noise input (Fig. S11 (c)). It becomes clear that both influence the scan result at transition parameter areas, where the behaviour of the system switches between no recruitment and full recruitment. In these transition areas, some degree of recruitment can be observed, depending on the noise input and the position of the stimulus.

B: Oscillatory microdomain on a bistable sheet

Fig. S12 investigates the relationship between $P_{Surrounding}$ and number of units in a contiguous microdomain. Similar to the previous case, the microdomain size required to elicit a full recruitment has to grow with decreasing $P_{Surrounding}$ (Fig. S12 (a)). Interestingly, when the sheet becomes monostable at $P = -2.5$, some large (about 30% of all units) microdomains can still elicit full recruitment. This is technically, a class III onset, as described in the main manuscript. We also show the variance of this scan result depending on the position of the stimulus (Fig. S12 (b)) and the noise input (Fig. S12 (c)). It becomes clear that both influence the scan result at transition parameter areas, where the behaviour of the system switches between no recruitment and full recruitment. In these transition areas, some degree of recruitment can be observed, depending on the position of the stimulus and in one case also on the noise input.

C: Oscillatory microdomains on a monostable sheet

Fig. S13 investigates the relationship between the number of oscillatory units and their spatial arrangement into subclusters on a monostable sheet. Fig. S13 (a) is the same as in the main manuscript Fig. 7 (a). Here, we additionally show the variance of this scan result depending on the position of the stimulus (Fig. S13 (b)) and the noise input (Fig. S13 (c)). It becomes clear that both influence the scan result at transition parameter areas, where the behaviour of the system switches between no recruitment and full recruitment. In these transition areas, some degree of recruitment can be observed, depending on the position of the stimulus and in one case also on the noise input.

D: Connectivity

Additionally, we have explored the effect of using different connectivity matrices (generated by the same algorithms and parameters, the only changes are due to the stochasticity of the algorithms). In the scans of recruitment from microdomains and stimuli, this effect is to an extent explored by different positioning of the microdomains and stimuli. Hence we demonstrate the effect of different connectivity matrices in a scan of the overall dynamics in the homogeneous system. Fig. S14 shows the variance of the overall dynamics of the stimulated system depending on P and Q. Again, we observe that some variation in the result occurs at transitions areas (in this case, between mono- and bistability). In the main manuscript we have used a single connectivity matrix throughout. This is justified as the connectivity matrix (produced by the same algorithm and parameters) does not affect the results. The exact parameter for onset of bistability could vary slightly with the connectivity matrix. However the classification of the global state of the simulated sheet into the background, bistable, and oscillatory state is not affected.

In summary, we demonstrated the existence of transition parameter regions in this section, where the recruitment or overall systems behaviour is sensitive to changes in position of microdomains, noise input,

or different connectivity matrices. This is not unexpected due to the heterogeneity in the connectivity and the stochasticity underlying its generation. However, we have demonstrated that the sensitivity to the underlying connectivity is only present in regions of a sudden qualitative change of behaviour (e.g. onset of bistability, onset "recruitability"). This variability emphasises our point of patient-specific derivation of cause and conditions of the seizures. E.g. a certain concentration, or density of microseizure domains does not necessarily cause seizures in one patient, but may lead to wide-spread recruitment in another patient, due to the differences in the affected area and its underlying connectivity. However, the classification we provided in the main manuscript are independent of specific parameter settings, but rather they categorise different dynamic conditions, which can occur in all patients in different patient-specific parameter settings.
