# Supplementary material for: Dynamic Mechanisms of Neocortical Focal Seizure Onset
Source: PLoS Comput Biol. 2014 Aug 14;10(8):e1003787. doi: 10.1371/journal.pcbi.1003787 (PMC4133160; doi:10.1371/journal.pcbi.1003787)
Supplement: Text S9 — Simulating seizure prevention. (PDF) [file pcbi.1003787.s026.pdf]

Text S9: Seizure prevention

From the theoretical perspective, we suggest two main types of treatment strategy: one to treat temporary external stimuli, and the other to treat prolonged intrinsic hyperactivity. If a global condition is causing or supporting seizure onset and recruitment, it is outside the scope of our work to suggest any treatment strategy, as the global conditions are not modelled mechanistically.

In the case of a spatially localised temporary perturbation/stimulus, we suggest that a counter-stimulus could be used to attenuate seizure activity, if applied early. Fig. S15 (a) shows a scenario in which a perturbation induces full recruitment of the system to the seizure state. In Fig. S15 (b) the same simulation is repeated (using the same noise vector) and a counter-stimulus is delivered at the time and position indicated in the figure. The application of the counter-stimulus is found to prevent the recruitment to seizure activity. Specifically the counter-stimulus suppresses firing activity in the excitatory population of the target units (which could be achieved clinically for example by using high frequency stimulation e.g. as in [**?**]). This method has the advantage that it would leave local tissue as well as their connectivity intact and only interacts with certain stimuli. However this is of course a suggestion from the theoretical perspective, we acknowledge that for practical usage the detection time is crucial and potentially only feasible for special cases. In terms of spatial accuracy, perhaps a spatially more extended counter stimulus can be used to ensure that the recruitment core is missed. Also after the seizure has started and developed, a broad counter stimulus could be used to abate the seizure early. An alternative would be to severe the connections transmitting the stimuli (if these are specific). E.g. in the case of a specific subcortical input, connections between the cortical region and the subcortical structure could be severed.

For cases in which seizure onset is initiated due to local, persistent hyperactivity, one way to stop seizure recruitment could be to destroy the integrity of the hyperactive part of the tissue. As demonstrated, the size of the hyperactive microdomain directly impacts its own activity and recruitment. In Fig. S16 we show an example of such a recruitment suppression. We start with the simulation of a hyperactive microdomain that results in onset and widespread distribution of seizure activity due to global bistability (Fig. S16 (a)). We then remove the connections that are intersected by one small cortical "cut" (red line) and repeat the simulation with the same noise vector. We find that the recruitment from hyperactive microdomains is weakened and the onset of widespread seizure activity delayed (Fig. S16 (b)). Finally, more extensive removal (a second cut) of connections leads to the restriction of abnormal activity to the microdomain (Fig. S16 (c)). No global spreading of abnormal dynamics is seen. Even after 20 s of simulation time, no recruitment is observed. In this example we have essentially separated the units in the hyperactive microdomain from each other by means of the connection removal, thereby reducing their influence on each other. This decreases their effective size and activity, and hence recruitment is first delayed and then suppressed altogether.
