# Supplementary material for: Dynamic Mechanisms of Neocortical Focal Seizure Onset
Source: PLoS Comput Biol. 2014 Aug 14;10(8):e1003787. doi: 10.1371/journal.pcbi.1003787 (PMC4133160; doi:10.1371/journal.pcbi.1003787)
Supplement: Text S10 — Effect of propagation delays. (PDF) [file pcbi.1003787.s027.pdf]

Text S10: Time delays

Signal propagation delays from one unit to another have not been incorporated in our model as they were not deemed to have any significant effect on the dynamics. At a signal propagation speed of 3 m/s, the delay time for the longest fibre (5.5 mm) is 1.83 ms, which is shorter than the simulation time step $2ms$ used for our system. Hence the system incorporating delays is numerically the same as the system without delays. At the mesoscopic scale, a signal propagation speed of $0.3m/s$ is realistic [1], if not faster, due to myelinated remote connection fibres [2–4]. Upon examination of the dynamics of the system using $0.3m/s$ propagation speed, the behaviour is still comparable. In fact the bifurcation behaviour is virtually the same (see Fig. S17 (a) and (b)). It is only when at $0.03m/s$, the bifurcation behaviour changes slightly (see Fig. S17 (c)), but still preserving the qualitative behaviour. Implicit delays are incorporated by the connectivity structure. Computationally, simulating the system with delays (at 0.3 m/s) also increases the computational time by an order of magnitude, which slows down the research process immensely. Hence, we simulate our systems in the manuscript without explicit delays.

References

1. Hirsch JA, Gilbert CD (1991) Synaptic physiology of horizontal connections in the cat's visual cortex. J Neurosci 11: 1800–1809.

2. Keller A, Asanuma H (1993) Synaptic relationships involving local axon collaterals of pyramidal neurons in the cat motor cortex. J Comp Neurol 336: 229–242.

3. DeFelipe J, Conley M, Jones EG (1986) Long-range focal collateralization of axons arising from corticocortical cells in monkey sensory-motor cortex. J Neurosci 6: 3749–3766.

4. Ruesch E (2011) Functional architecture of superficial layer pyramidal neurons in the cat primary visual cortex. Ph.D. thesis, ETH Zuerich. URL `http://dx.doi.org/10.3929/ethz-a-006499154`.
